# The L-lactate dehydrogenases of *Pseudomonas aeruginosa* are conditionally regulated but both contribute to survival during macrophage infection

**DOI:** 10.1128/mbio.00852-24

**Published:** 2024-08-20

**Authors:** Lindsey C. Florek, Xi Lin, Yu-Cheng Lin, Min-Han Lin, Arijit Chakraborty, Alexa Price-Whelan, Liang Tong, Laurence Rahme, Lars E. P. Dietrich

**Affiliations:** 1 Department of Biological Sciences, Columbia University, New York, New York, USA; 2 Department of Dentistry, National Yang Ming Chiao Tung University, Taipei, Taiwan; 3 Department of Surgery, Massachusetts General Hospital, and Harvard Medical School, Boston, Massachusetts, USA; 4 Shriners Hospitals for Children Boston, Boston, Massachusetts, USA; 5 Department of Microbiology, Harvard Medical School, Boston, Massachusetts, USA; Geisel School of Medicine at Dartmouth, Hanover, New Hampshire, USA

**Keywords:** lactate, biofilm, *Pseudomonas aeruginosa*, glycolate, macrophages

## Abstract

*Pseudomonas aeruginosa* is an opportunistic pathogen that thrives in environments associated with human activity, including soil and water altered by agriculture or pollution. Because L-lactate is a significant product of plant and animal metabolism, it can serve as a carbon source for *P. aeruginosa* in the diverse settings that it inhabits. In this study, we evaluate the production and use of two redundant *P. aeruginosa* L-lactate dehydrogenases, termed LldD and LldA. We confirm that the protein LldR represses *lldD* and identify a new transcription factor, called LldS, that activates *lldA*; these distinct regulators and the genomic contexts of *lldD* and *lldA* contribute to their differential expression. We demonstrate that the *lldD* and *lldA* genes are conditionally controlled in response to lactate isomers as well as to glycolate and ɑ-hydroxybutyrate, which, like lactate, are ɑ-hydroxycarboxylates. We also show that *lldA* is induced when iron availability is low. Our examination of *lldD* and *lldA* expression across depth in biofilms indicates a complex pattern that is consistent with the effects of glycolate production, iron availability, and cross-regulation on enzyme preference. Finally, macrophage infection assays reveal that both *lldD* and *lldA* contribute to persistence within host cells, underscoring the potential role of L-lactate as a carbon source during *P. aeruginosa–*eukaryote interactions. Together, these findings help us understand the metabolism of a key resource that may promote *P. aeruginosa*’s success as a resident of contaminated environments and animal hosts.

**IMPORTANCE:**

*Pseudomonas aeruginosa* is a major cause of lung infections in people with cystic fibrosis, of hospital-acquired infections, and of wound infections. It consumes L-lactate, which is found at substantial levels in human blood and tissues. In this study, we investigated the spatial regulation of two redundant enzymes, called LldD and LldA, which enable L-lactate metabolism in *P. aeruginosa* biofilms. We uncovered mechanisms and identified compounds that control the preference of *P. aeruginosa* for LldD versus LldA. We also showed that both enzymes contribute to its ability to survive within macrophages, a behavior that is thought to augment the chronicity and recalcitrance of infections. Our findings shed light on a key metabolic strategy used by *P. aeruginosa* and have the potential to inform the development of therapies targeting bacterial metabolism during infection.

## INTRODUCTION

Lactate is a small organic compound and a metabolite that is present in diverse environments. In the rhizosphere or within animal hosts, lactate can constitute a major carbon source for commensal and pathogenic microbes (1–4). The bacterial “NAD-independent” lactate dehydrogenases, referred as “iLDHs,” enable growth on lactate by oxidizing it to pyruvate (5). iLDHs are specific for either the D- or L-isomer of lactate, and bacteria generally show variation in their complements of these enzymes (6). Some bacteria also show iLDH redundancy, i.e., the presence of more than one enzyme that can act on a given isomer, which is particularly prevalent among species that produce iLDHs that act on L-lactate (“L-iLDHs”) (File S1) (6). This type of redundancy can make biological sense if redundant genes differ in their spatial or temporal regulation and are thus specialized for distinct environmental contexts (7–10).

**Fig 1:**
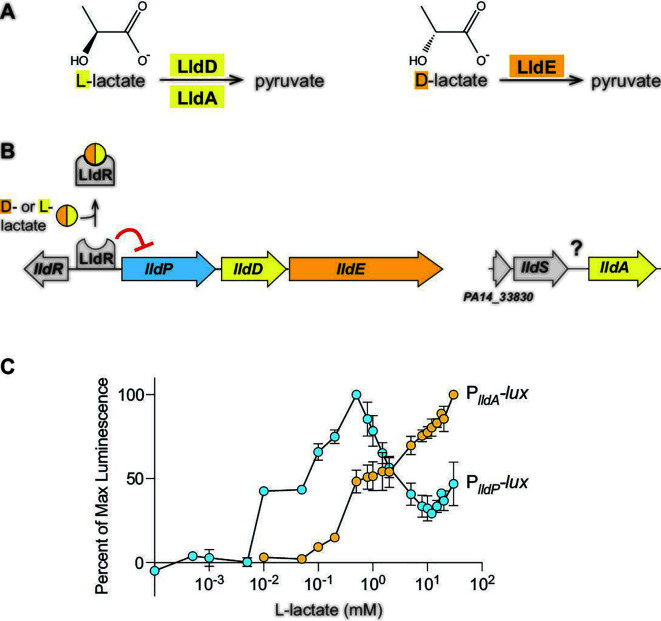
Genes for *P. aeruginosa* L-lactate dehydrogenases are sensitive to different lactate isoforms and concentrations. (**A**) Reactions carried out by the L-lactate dehydrogenases (LldD; LldA) and the D-lactate dehydrogenase (LldE). (**B**) Chromosomal arrangement of genes associated with lactate utilization in *P. aeruginosa*. The *lldR* gene encodes a transcriptional repressor for the *lldPDE* operon, while details regarding the regulation of *lldA* are unknown. (**C**) Activities of the *lldA* and *lldPDE* promoters at L-lactate concentrations ranging from 0.1 µM to 30 mM. Cultures of luminescent reporter strains (P*_lldA_-lux* and P*_lldP_-lux*) were grown with shaking in a 96-well plate at 37°C for 24 hours in a base medium buffered with 3-(N-morpholino) propanesulfonic acid (MOPS) and containing 20 mM succinate. Each value shown represents the maximum luminescence produced during growth in the indicated L-lactate concentration, normalized to the maximum luminescence value produced in the most stimulatory L-lactate concentration. Values shown for each concentration are averages of two biological replicates, and error bars represent standard deviation.

Bacteria of the genus *Pseudomonas* are found in aquatic and terrestrial settings and are common colonizers of eukaryotic hosts (11). One outstanding feature of pseudomonads is their ability to use a wide range of carbon sources and their preferential use of organic acids, including lactate, over sugars (12). The opportunistic pathogen *P. aeruginosa* is a common cause of hospital-acquired infections and infections in immunocompromised individuals (13). *P. aeruginosa* is able to grow using both D- and L-lactate, and our group has previously shown that it produces functionally redundant L-iLDHs (Fig. 1A) (14). Both LldD and LldA are predicted to bind a flavin mononucleotide (FMN) cofactor and to couple L-lactate oxidation to reduction of the quinone pool (15). The maintenance of redundant L-iLDHs in *P. aeruginosa*, which is unusual among the pseudomonads in that it can thrive in natural environments and in human infection sites (16), raises the question of whether context-dependent production of L-iLDHs contributes to its adaptability.

In addition to its metabolic versatility and ability to colonize humans, *P. aeruginosa* is notorious for its formation of biofilms: aggregates of microbial cells encased in a self-produced and protective matrix (17). These multicellular structures are well-suited to the study of condition-dependent gene expression because they contain steep chemical gradients that lead to the formation of microniches and metabolically differentiated subpopulations (18). Prior observations by our group have suggested that *P. aeruginosa lldD* is heterogeneously expressed in laboratory-grown biofilms (14, 19), while a study that applied a machine learning approach to transcriptomic data identified *lldA* as a locus specifically associated with human infection (20). In this work, we set out to identify conditions and mechanisms that control L-lactate dehydrogenase gene expression in *P. aeruginosa*. Our findings illustrate the complexity of redundant gene utilization, which has consequences for *in vitro* growth and facilitates bacterial survival during infection. Moreover, our study results show that differential regulation of redundant genes translates into physiological heterogeneity during multicellular *P. aeruginosa* growth. This work provides insight into context-dependent differentiation that may contribute to *P. aeruginosa*’s success as a prominent cause of biofilm-based and chronic infections.

## RESULTS

### *lldPDE* and *lldA* are differentially expressed in response to isomer identity and L-lactate concentration

The genes for the L-lactate dehydrogenases LldD and LldA are situated at distinct sites on the *P. aeruginosa* chromosome. *lldD* is located within an operon that also codes for a lactate permease (LldP) and a D-lactate dehydrogenase (LldE), while *lldA* is monocistronic (Fig. 1A and B). Previously, we showed that both lactate isomers induce *lldPDE* expression, while only L-lactate induces *lldA* expression, and that *lldPDE* and *lldA* exhibit different expression dynamics in liquid cultures (14). These results are consistent with the idea that differential expression might allow the redundant L-iLDHs to contribute to fitness under distinct environmental conditions. Accordingly, we sought to examine the parameters, and investigate the roles of potential regulators, that might differentially affect *lldD* and *lldA* expression.

First, we asked if *lldD* and *lldA* are induced by different L-lactate concentrations. To address this question, we engineered the reporter strains P*_lldP_-lux* and P*_lldA_-lux*, which express the *luxCDABE* operon under control of the indicated promoters (21). *lux*-based reporter constructs produce the luciferase enzyme (LuxAB) as well as its substrate (via LuxCDE) and have increased sensitivity to promoter activity compared to fluorescent protein-based reporters (22). In the P*_lldP_-lux* and P*_lldA_-lux* strains, the constructs have been cloned into a neutral site on the chromosome; each luciferase signal serves as a readout for promoter activity and therefore reports *lldPDE* or *lldA* expression. We grew planktonic cultures of the P*_lldP_-lux* and P*_lldA_-lux* reporter strains with 20 mM succinate and various concentrations of L-lactate ranging from 0.1 µM to 30 mM. We detected luminescence from P*_lldP_-lux* expression already at 10 µM L-lactate, while that of P*_lldA_-lux* required 10 x higher concentrations. In contrast to P*_lldA_-lux*, whose expression positively correlated with the L-lactate concentration, P*_lldP_-lux* expression peaked at 500 µM L-lactate (Fig. 1C). These findings indicate that the expression of *lldPDE* and *lldA* is fine-tuned to different L-lactate concentrations and suggest distinct regulatory mechanisms.

### LldS is required for *lldA* expression

Although previous studies have identified the transcriptional repressor LldR, which controls the expression of *lldPDE* (23), regulators of *P. aeruginosa lldA* expression have not yet been described. To test whether LldR impacts *lldA* expression, we deleted *lldR* in a P*_lldA_-gfp* reporter strain (which contains a P*_lldA_*-driven GFP expression construct cloned at a neutral site on the chromosome) and grew this strain in a liquid medium containing L-lactate. Deletion of the *lldR* gene had only a modest effect on P*_lldA_* activity (Fig. S1), indicating that LldR is not a major regulator of *lldA* expression and supporting our finding that *lldA* and *lldD* are differentially regulated (Fig. 1C).

**Fig 2:**
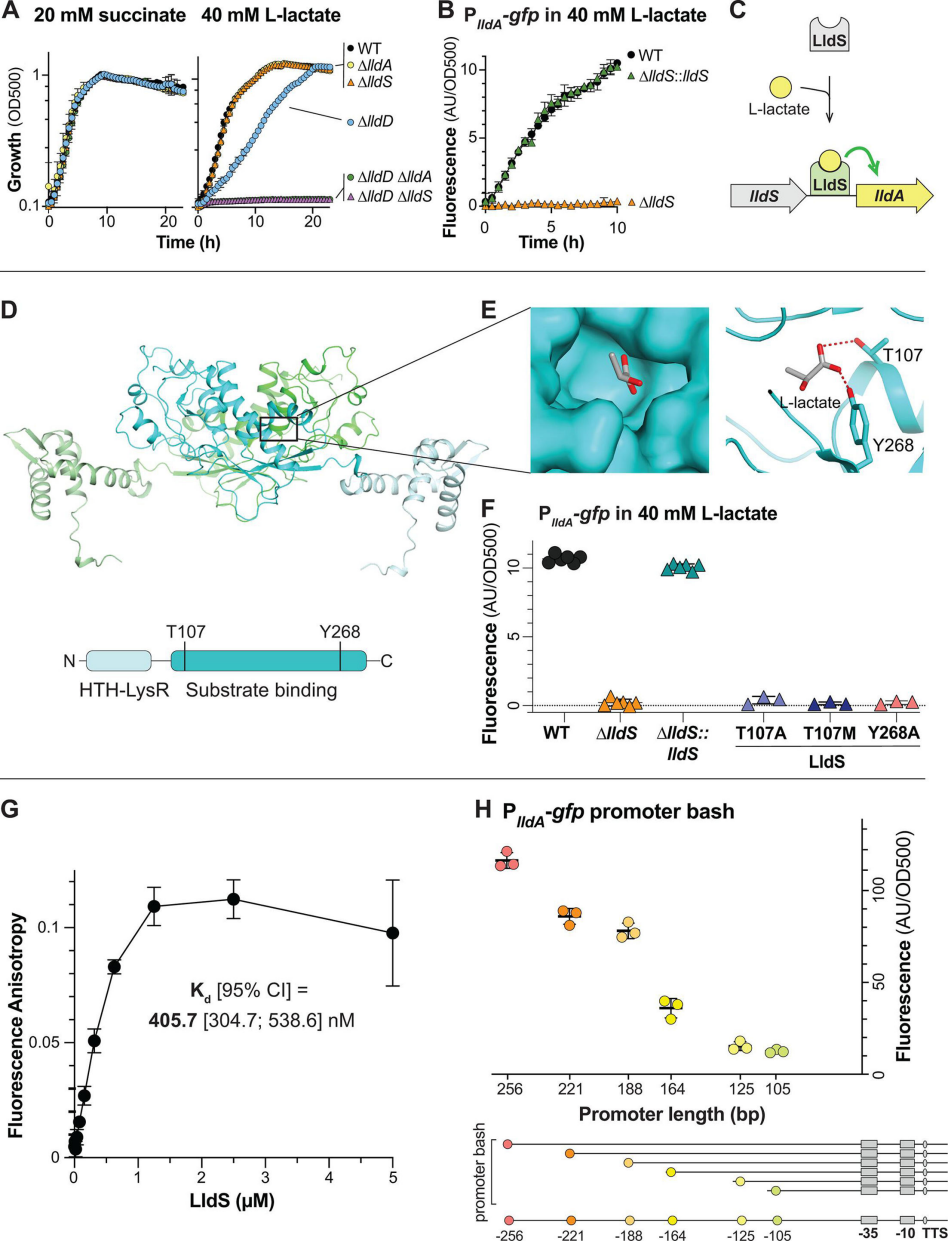
LldS (PA14_33840) is necessary for expression of *lldA* and likely senses L-lactate via its ligand-binding domain. (**A**) Growth of wild-type (WT) and mutants, lacking various genes associated with lactate metabolism, as liquid cultures in MOPS medium containing 20 mM succinate (left) or 40 mM L-lactate (right) as the sole carbon source. (**B**) *lldA* promoter activity in liquid cultures of WT, ∆*lldS*, and the ∆*lldS* complementation strain grown in MOPS medium containing 40 mM L-lactate. We observe that growth of ∆*lldS* under this condition is supported by LldD, as indicated by the results in panel **A**. (**C**) Schematic of the proposed mechanism regulating *lldA* expression. (**D**) (Top) AlphaFold-predicted structure of the LldS dimer with the individual monomers colored green and cyan and with lighter shades representing the DNA-binding domains. (Bottom) Domain architecture of the LldS protein. (**E**) Left: molecular surface of the predicted LldS binding pocket, containing L-lactate. Right: ribbon model of the predicted LldS binding pocket with two residues, T107 and Y268, shown interacting with L-lactate. (**F**) *lldA* promoter activity in liquid cultures of ∆*lldS* strains complemented with wild-type *lldS* or with the LldS point mutants T107A, T107M, and Y268A. Strains were grown in MOPS medium containing 40 mM L-lactate. Fluorescence values were taken 5–6 hours after the onset of the stationary phase. Data points represent biological replicates, and error bars represent standard deviation. (**G**) Binding curve of LldS to a 5’FAM-labeled DNA probe containing 256 bp upstream of the start codon of *lldA*. Protein concentrations ranged from 9.3 nM to 5 µM, and the probe concentration was 5 nM. The calculated K_d_ value is shown, and error bars represent standard deviation of two to three replicates per concentration. (**H**) Top: fluorescence of P*_lldA_-gfp* reporter strains with promoter regions of the indicated length. Each value was normalized by subtracting the average background fluorescence value of ∆*lldS* containing the full-length promoter construct. Cultures were incubated for 15 hours in MOPS medium containing 40 mM L-lactate. Data points represent biological replicates, and error bars represent standard deviation. Bottom: diagram depicting the truncations made for “promoter bash” constructs. The predicted −10 and −35 boxes and transcription start site (TSS) are indicated. These motifs were identified using the SAPPHIRE tool (27). For plots shown in panels A and B, error bars represent the standard deviation of biological triplicates and are obscured by the point marker in some cases.

In bacterial genomes, including that of *P. aeruginosa*, it is common for the gene encoding a transcriptional regulator to lie adjacent to a target of the regulator (24). The gene just upstream of *lldA*, which we have named *lldS* (Fig. 1B), encodes a regulatory protein predicted to contain a LysR-type substrate-binding domain and a DNA-binding domain (25). LysR-type transcription factors have been implicated in the regulation of lactate utilization in other bacteria (6, 26). To test whether LldS affects *lldA* expression, we deleted *lldS* (*PA14_33840*) in a ∆*lldD* mutant. This strain was unable to grow when L-lactate was provided as the sole carbon source, recapitulating the phenotype of a ∆*lldD* ∆*lldA* mutant (14), but did not show a growth defect when succinate was provided as the sole carbon source (Fig. 2A). Moreover, we deleted *lldS* in the P*_lldA_-gfp* reporter strain background and found that this abolished P*_lldA_* activity in the presence of L-lactate (Fig. 2B). Chromosomal complementation (insertion of the wild-type *lldS* allele at the native site in ∆*lldS*) restored *lldA* expression to wild-type levels. Together, these observations suggest that *lldS* encodes a transcriptional regulator that directly activates P*_lldA_* expression in response to L-lactate (Fig. 2C).

To further evaluate the potential of LldS as an L-lactate-dependent transcriptional activator, we used AlphaFold2 (28) to predict its structure and identified a potential L-lactate binding pocket based on the structure of an LldS homolog in complex with a peptide ligand that has a D-alanine at the C-terminus (PDB 4WKM) (29) (Fig. 2D). This pocket contains two residues that might coordinate the carboxyl group of L-lactate, T107 and Y268 (Fig. 2E), which are equivalent to the interactions for the carboxyl group of D-alanine. To test whether these residues contribute to L-lactate-dependent expression of *lldA*, we generated the LldS point mutants T107A, T107M, and Y268A. We found that all three mutations prevented the activation of P*_lldA_* in response to L-lactate (Fig. 2F). While it is possible that these mutations abolished *lldA* expression by yielding unstable variants, the fact that the residue changes are expected to have diverse chemical and structural effects supports the interpretation that their impact on gene expression arises from defects in substrate binding. Specifically, when paired with AlphaFold2 modeling results, our observations suggest that conversion of the polar threonine and tyrosine residues to alanine interferes with hydrogen bonding to L-lactate and that the conversion of threonine to methionine sterically hinders L-lactate in the binding pocket.

### LldS binds the *lldA* promoter sequence

To examine the interaction between LldS and the sequence upstream of *lldA*, we purified 6×-histidine-tagged *P. aeruginosa* PA14 LldS protein from *Escherichia coli* and assayed its binding to a 5′-fluorophore-labeled DNA composed of the 256-bp sequence upstream of *lldA* (i.e., the putative *lldA* promoter region) using a fluorescence polarization experiment. We generated binding curves by incubating 5 nM of the DNA with LldS at concentrations ranging from 9.37 nM to 5 µM and determined *K*_*d*_ values for the DNA–protein interaction (Fig. 2G). We calculated an average *K*_*d*_ (95% CI) value of 405.7 (304.7; 538.6) nM for LldS probe binding.

Having found that LldS binds to the 256-bp sequence upstream of *lldA*, we sought to further narrow down the sequence required for *lldA* expression. We therefore characterized the region upstream of this gene using a “promoter bash” approach (30). P*_lldA_-gfp* reporter strains with promoters of different lengths (256, 221, 188, 164, 125, and 105 bp) were grown as liquid cultures in MOPS medium containing L-lactate. We observed a gradual decrease in P*_lldA_-gfp* activity with decreasing promoter length. However, we observed the most pronounced difference in P*_lldA_-gfp* activity between the constructs that contained 164 versus 188 bp of the sequence upstream of the start codon, which may indicate that a transcription factor binds in this region (Fig. 2H).

### *lldPDE* and *lldA* respond differentially to ɑ-hydroxycarboxylates

To test whether metabolites other than D- or L-lactate affect P*_lldP_* or P*_lldA_* activity, we screened 95 compounds using the phenotype microarray plate PM1 (Biolog, Inc.) (File S2). A dual-fluorescent transcriptional reporter strain, containing the *lldA* promoter driving *mScarlet* expression and the *lldPDE* promoter driving *gfp* expression, was used for these screens (Fig. S2). In this reporter strain, the constructs have been cloned into a neutral site on the chromosome; each fluorescence signal serves as a readout for promoter activity and therefore reports *lldPDE* or *lldA* expression. To screen for activating compounds, we tested for increased expression in a base medium containing succinate as the sole carbon source. For *lldPDE* expression, we did not identify any activating compounds other than L-lactate (plate PM1 does not contain D-lactate). For *lldA* expression, we found that, in addition to L-lactate, α-hydroxybutyrate (α-HB) had a significant stimulatory effect (Fig. S2). To screen for inhibitory compounds, we tested for decreased expression in a base medium containing succinate and L-lactate. For *lldA* expression, we did not identify any inhibitory compounds, but for *lldPDE* expression, we found that glycolate, which differs from lactate by the removal of one methyl group, had a strong inhibitory effect.

**Fig 3:**
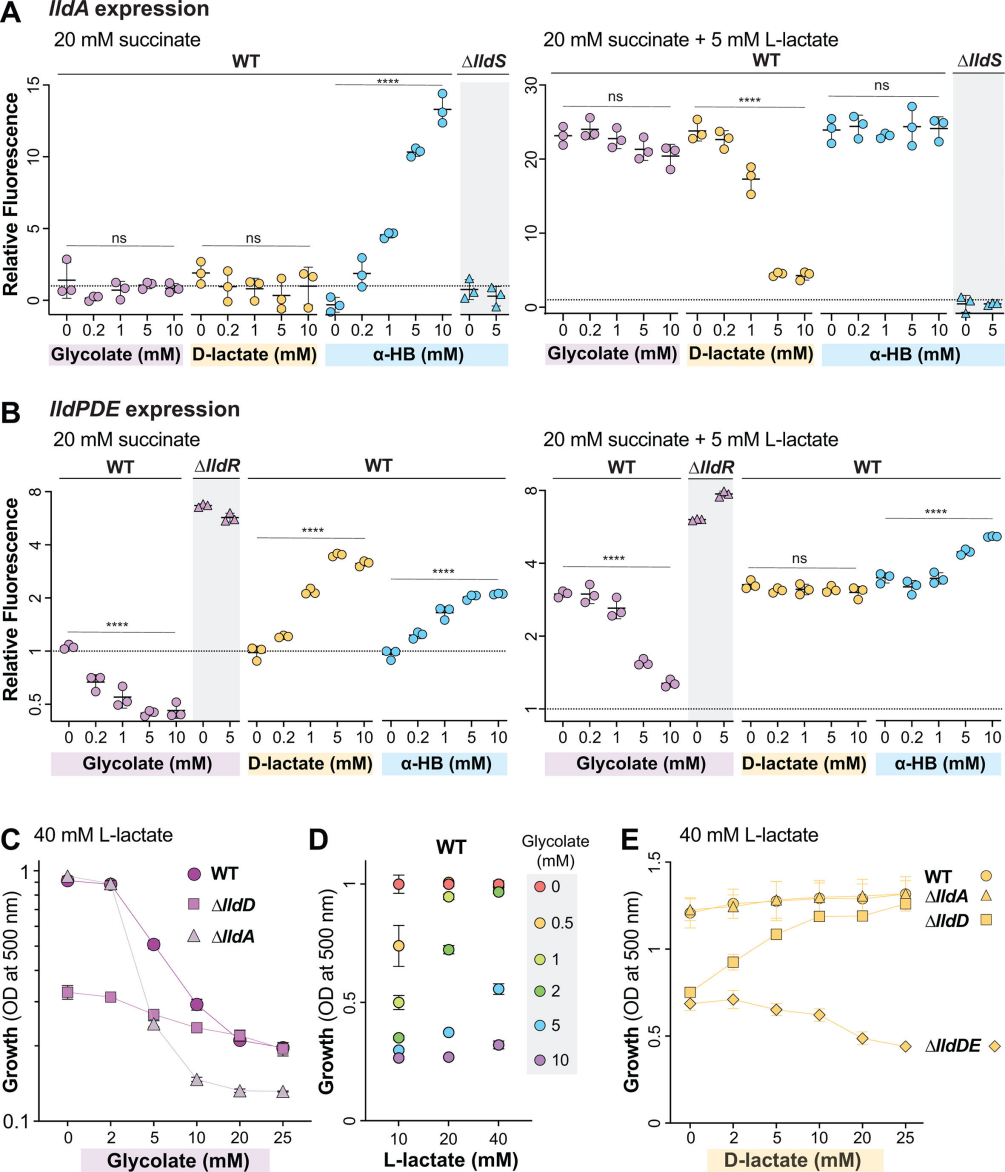
Identification of additional effectors of lactate gene regulation. (**A, B**) Fluorescence values 5–6 hours after the onset of the stationary phase produced by the *lldA* and *lldPDE* reporter strains. The dashed line represents fluorescence of a wild-type culture grown with 20 mM succinate, and fluorescence values are expressed in relation to this value. Cultures were grown in liquid MOPS medium containing 20 mM succinate (left graphs) or 20 mM succinate +5 mM L-lactate (right graphs) and amended with the indicated compounds. Fluorescence values of mutant strains (∆*lldS* for the *lldA* graphs and ∆*lldR* for the *lldPDE* graphs) are also shown for select compounds, as indicated. Each dot is representative of a biological replicate, and error bars represent standard deviation. **** indicates a *P*-value < 0.0001; ns = not significant. (**C**) Growth at 10 hours of WT, ∆*lldD*, and ∆*lldA* in MOPS medium containing 40 mM L-lactate with glycolate concentrations ranging from 0 to 25 mM. (**D**) Growth of WT in MOPS medium containing L-lactate concentrations of 10, 20, or 40 mM with added glycolate concentrations ranging from 0 to 10 mM. Measurements were taken at the time point when the 0 mM glycolate condition for each L-lactate concentration reached the stationary phase. Values shown are relative to growth yield at 0 mM glycolate for each respective L-lactate concentration. (**E**) Growth at 15 hours of WT, ∆*lldD*, ∆*lldA*, and ∆*lldDE* strains grown in MOPS medium containing 40 mM L-lactate, with D-lactate concentrations ranging from 0 to 25 mM.

To verify the screen results, we surveyed the effects of α-HB, glycolate, or D-lactate on *lldA* (Fig. 3A) and *lldPDE* expression (Fig. 3B) by adding the compounds at concentrations ranging from 0.2 to 10 mM to a base medium containing either succinate alone or succinate in combination with L-lactate. In cultures grown in a base medium containing succinate, we confirmed that α-HB was the only compound of the three that affected *lldA* expression and found that this effect was abolished in the ∆*lldS* background. This suggested that LldS activity might be affected by both L-lactate and α-HB; we therefore examined the activity of the more-sensitive P*_lldA_-lux* reporter in media containing a range of concentrations of each compound and found that the promoter is more sensitive to L-lactate (Fig. S3). *lldPDE* expression, meanwhile, was affected by all three surveyed compounds: D-lactate showed the expected stimulatory effect, α-HB showed a moderate stimulatory effect, and glycolate slightly inhibited even background expression. This background expression is the residual expression detected in the absence of an exogenous stimulant and may arise from endogenous production of D-lactate. (*P. aeruginosa* can produce D-lactate via the enzyme LdhA [31]; it does not produce L-lactate.) The glycolate effect is likely to be LldR-mediated, as its inhibitory function is lost in a ∆*lldR* strain (Fig. 3B), which has high lactate-independent *lldPDE* expression.

In the “stimulatory” base medium containing both succinate (20 mM) and L-lactate (5 mM), the addition of glycolate had no significant effect on *lldA* expression. In contrast, increasing amounts of D-lactate had an inhibitory effect on *lldA* expression, beginning at 1 mM. We also observed that in the stimulatory base medium, α-HB had no effect on *lldA* expression. This finding is consistent with our study results, suggesting that P*_lldA_* is more sensitive to L-lactate than α-HB (Fig. S3) and indicates that α-HB is not a strong competitor for LldS binding in the presence of L-lactate. With respect to *lldPDE* expression, we found that glycolate showed an inhibitory effect even in the presence of L-lactate, particularly when it was added at an equimolar concentration. D-lactate did not stimulate *lldPDE* expression beyond the level arising from L-lactate-dependent induction. Finally, α-HB showed a slight stimulatory effect comparable to that observed in the medium without lactate.

Overall, the results of these experiments show that the activities of P*_lldP_* and P*_lldA_* are differentially affected by various α-hydroxy acid compounds. While *lldPDE* expression is stimulated by D-lactate and inhibited by glycolate, *lldA* expression is inhibited by D-lactate and relatively unaffected by glycolate. The effect of glycolate on P*_lldP_* is mediated by the repressor LldR (Fig. 3B). The effect of D-lactate on P*_lldA_* could indicate that LldS, which is activated by L-lactate, is deactivated by the D-isomer of lactate. An alternative explanation, however, is that D-lactate appears to inhibit L-lactate-dependent *lldA* expression because less L-lactate enters the cell when both isomers are provided simultaneously due to competition for the lactate permease (32). Finally, both promoters are activated by α-HB, but in the case of P*_lldA_*, this requires the regulator LldS (Fig. 3A), while for P*_lldP_*, we do not yet have an indication as to how this activation is mediated.

Consistent with the observations that LldD is the primary contributor to growth on L-lactate under standard conditions (Fig. 2A) (14) and that *lldPDE* expression is inhibited by glycolate (Fig. 3B), previous study results have indicated that *P. aeruginosa* growth on lactate is inhibited by the addition of glycolate (23, 33). This raised the question of whether growth of *P. aeruginosa* on L-lactate plus glycolate increases its reliance on LldA, whose expression is unaffected by glycolate (Fig. 3A). To test this, we grew PA14 WT alongside ∆*lldD* and ∆*lldA* mutants in 40 mM L-lactate with added glycolate concentrations ranging from 0 to 25 mM (Fig. 3C). As expected, we found that glycolate inhibited wild-type growth, causing a sharp decrease in growth yield at 5 mM glycolate. In contrast, although ∆*lldD* showed decreased growth consistent with the LldD’s primary role in L-lactate utilization, the effect of increasing glycolate concentrations on this yield was less pronounced and was indistinguishable from the effect of WT at concentrations of 20 mM and higher. The residual growth of WT and ∆*lldD* at high glycolate concentrations indicates LldD-independent L-lactate utilization that is less sensitive to glycolate. Accordingly, in contrast to ∆*lldD*, the ∆*lldA* mutant showed a pronounced decrease in growth yield at glycolate concentrations of 5 mM and higher when compared to the WT. These results show that LldA makes an important contribution to growth of *P. aeruginosa* on L-lactate in the presence of glycolate. Finally, to test whether sensitivity to glycolate is affected by the concentration of L-lactate provided, we performed similar experiments with a base L-lactate concentration of 10 or 20 mM for a direct comparison with the effects of 40 mM L-lactate on growth (Fig. 3D). We found that cultures grown on lower concentrations of L-lactate were sensitive to lower concentrations of glycolate, with glycolate negatively affecting growth starting at only 0.5 mM glycolate in the 10 mM-L-lactate culture and at 2 mM glycolate in the 20 mM-L-lactate culture. This observation suggests that glycolate might competitively bind to LldR and enhance its repression of *lldPDE* expression.

In addition to the negative effect of glycolate on P*_lldP_* activity, our expression analysis revealed an inhibition of P*_lldA_* activity by D-lactate (Fig. 3A). We hypothesized that this would lead to an increased reliance on LldD under conditions where both lactate isomers were present. We therefore predicted that addition of D-lactate would be detrimental to the growth of the ∆*lldD* mutant on L-lactate. We grew WT *P. aeruginosa* alongside ∆*lldD* and ∆*lldA* mutants in 40 mM L-lactate with added D-lactate concentrations ranging from 0 to 25 mM. We found that added D-lactate had no effect on WT and ∆*lldA* growth. Unexpectedly, however, it had a stimulatory effect on ∆*lldD* growth (Fig. 3E). We reasoned that this stimulation arose from the utilization of D-lactate via LldE activity (Fig. 1A) and therefore repeated the experiment, this time including a ∆*lldDE* mutant. As expected, this mutant showed decreased growth in D-lactate concentrations of 10 mM or higher.

### The genomic context of *lldA* suggests a link to iron availability

**Fig 4:**
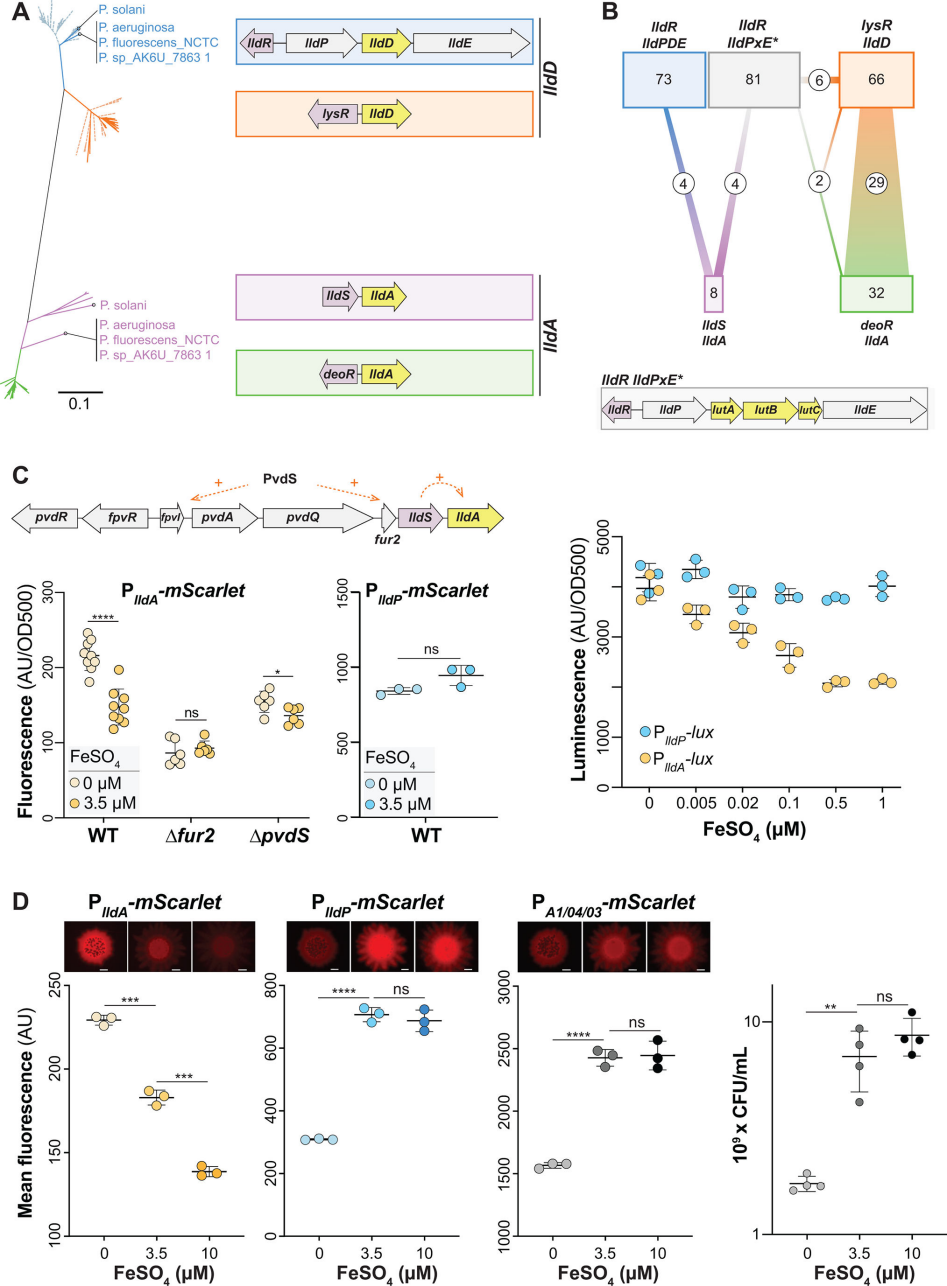
*lldA*, but not *lldPDE*, expression is sensitive to iron availability. (**A**) Phylogenetic tree of *lldD* and *lldA* genes obtained from 213 pseudomonad genomes. Only one representative strain per species was included, unless strains showed different *lld* arrangements. Blue and orange lines represent genes with homology to *lldD,* while green and purple lines represent genes with homology to *lldA*. Dotted lines indicate strains with one L-iLDH; solid lines indicate strains with more than one L-iLDH. The color of each cluster corresponds to the outlines surrounding the gene arrangement patterns shown on the right. Species/strain name is written if the strain has two L-iLDH genes. Only eight *Pseudomonas* species (including *P. aeruginosa*) possess the *lldS–lldA* gene arrangement outlined in purple. (**B**) Diagram depicting genome associations for the arrangement patterns shown (**A**) (indicated by a consistent color coding). An additional gene locus with the L-lactate utilization genes *lutABC*, which is not reflected in the tree, is outlined in gray and depicted at the bottom of this panel. The number of species possessing each gene arrangement is indicated in its corresponding box, and the connections represent the number of species with multiple L-iLDHs and their corresponding gene arrangements. (**C**) Top: in *P. aeruginosa*, *lldS–lldA* lies directly downstream of several genes involved in iron regulation and uptake. Left: *lldA* promoter activity in liquid cultures of WT, ∆*fur2*, and ∆*pvdS* strains grown in MOPS medium containing 40 mM L-lactate with ferrous sulfate added (3.5 µM) or no iron added (0 µM). Fluorescence values were taken 7 hours into the growth curve. Center: *lldPDE* promoter activity under the same conditions used for the P*_lldA_* reporter. Right: *lldA* and *lldPDE* promoter activity assayed using luciferase reporters. Cultures were grown in MOPS liquid medium with 40 mM L-lactate, and ferrous sulfate was added as indicated. The maximum luminescence value for each condition is shown for each reporter. (**D**) Representative top-down fluorescent biofilm images and quantification of the average fluorescence across the width of the center of the biofilm for the *lldA* reporter (left), *lldPDE* reporter (center left), and constitutive *mScarlet* (center right) strains. Biofilms were grown on MOPS medium with 20 mM succinate and 10 mM L-lactate, amended with ferrous sulfate as indicated. Scale bars = 2 mm. Right: quantification of colony-forming units (CFUs) of biofilms grown under each iron-amendment condition. Each dot is representative of a biological replicate, and error bars represent standard deviation. **P < 0.01, ***P < 0.001, and ****P < 0.0001; ns = not significant.

Having observed effects of organic metabolites and local regulators on the expression of *P. aeruginosa*’s redundant L-iLDH genes, we next examined the phylogenetic relationship of these proteins for clues regarding their respective physiological roles. Instead of or in addition to LldD and/or LldA, some organisms possess an L-iLDH encoded by a three-gene cluster referred to as *lutABC* (34). We searched for *lldD*, *lldA,* and *lutABC* homologs in *Pseudomonas* genomes from the Pseudomonas Genome DB (35), choosing one representative strain genome for each *Pseudomonas* species. Out of the 213 strains with L-iLDHs, we identified LldD and LldA homologs in 179 of these genomes. Fig. 4A shows a phylogenetic tree generated using the corresponding sequences and depiction of their gene arrangements. File S1 lists all 213 analyzed strains and indicates their L-iLDH profiles; those containing LldA or LldD are arranged according to the Lld phylogeny. Fig. 4B shows the total numbers of analyzed genomes that contain each of the indicated L-iLDH gene arrangements and the numbers of genomes that contain two or more L-iLDH homologs. In the tree shown in Fig. 4A, the *lldD* and *lldA* homologs formed independent clusters. Each of these clusters, however, also contained subgroupings that largely correlated with the genomic neighborhood/arrangement of *lldD* and *lldA* genes. *lldD* genes separated into two main subgroups: (i) those contained within an *lldPDE* operon, adjacent to a divergently transcribed *lldR* homolog (indicated in blue; as in *P. aeruginosa* PA14), and (ii) those that were monocistronic, located next to a divergently transcribed gene for a LysR-family transcriptional regulator (indicated in orange) (Fig. 4A).

*lldA* homologs showed a more scattered phylogeny overall but also formed two subgroups with consistent genomic arrangements: (i) a monocistronic *lldA* gene located next to a divergently transcribed gene for the DeoR-family transcriptional regulator (indicated in green) and (ii) a much less common *lldA* gene located directly downstream of an *lldS* homolog (indicated in purple) (Fig. 4A and B). The *P. aeruginosa lldA* sequence fell within the latter subgroup. *P. aeruginosa*, however, stood out as the only species in which *lldS* and *lldA* are situated near a large chromosomal region involved in the uptake of iron (Fig. 4C). *PA14_33830* (“*fur2*”), which lies upstream of *lldS* and bears homology to the global iron regulator Fur, is highly upregulated under low iron availability (36, 37) and has been implicated in the regulation of a wide range of genes, including those involved in iron uptake and siderophore production (38).

### Expression of *lldA* is enhanced under low-iron conditions

Given the proximity of *lldS* and *lldA* to the cluster of iron-related genes, we tested the effect of iron availability on *lldA* expression. The defined medium that we typically use for *P. aeruginosa* growth contains iron added as freshly prepared ferrous sulfate at a concentration of 3.5 µM. We grew the P*_lldA_-mScarlet* strain in this liquid medium with L-lactate as the carbon source, with or without added iron. Because iron chelators were not added to the “low-iron” medium, residual amounts of contaminating iron enabled *P. aeruginosa* growth (albeit at levels that were significantly lower than that observed for the standard (i.e., iron-amended or -replete) medium) (Fig. S4A). We found that OD-corrected *lldA* expression was enhanced under the low-iron condition when compared to the expression in the standard medium (Fig. 4C, left). This effect was not observed for *lldPDE* expression (Fig. 4C, center). While here only a single time point is shown, we also provide the full-time course data in Fig. S4B. Increasing the concentration of added iron to 10 µM also had no effect on *lldA* expression under these (liquid culture) conditions (Fig. S5). To better visualize subtle effects on gene expression that might arise under various conditions of iron availability, we once again utilized the luciferase reporters (P*_lldA_-lux* and P*_lldP_-lux*) and tested concentrations of ferrous iron sulfate ranging from 5 nM to 1 µM. While *lldA* expression decreased in a stepwise fashion with increasing iron concentrations, we did not observe significant changes in *lldPDE* expression (Fig. 4C, right).

How is the effect of iron on *lldA* expression mediated? To address this question, we tested the contributions of two regulatory genes, *fur2* and *pvdS*, to *lldA* expression under conditions of added or omitted ferrous iron sulfate. PvdS is a sigma factor that directly controls the expression of several loci in the region adjacent to *lldS*, including *fpvI* (Fig. 4C), and that has been implicated in the regulation of *lldS* (36, 39). We found that, in both the ∆*fur2* and ∆*pvdS* strain backgrounds, the enhancement of *lldA* expression observed in the WT under low-iron conditions was abolished (Fig. 4C). These results suggest that both Fur2 and PvdS contribute to the iron-dependent effects on *lldA* expression.

Nutrient limitation is a key determinant of the physiological status in biofilms because multicellularity promotes chemical gradient formation (18). We therefore tested the effect of iron availability on *lldA* and *lldPDE* expression in biofilms. Our group studies the physiology of bacteria in biofilms using a macrocolony assay, in which a suspension of cells is pipetted onto an agar-solidified growth medium and incubated in a standard atmosphere at high humidity for several days (40, 41). To examine the effects of iron availability on *lld* expression in biofilms, we grew the P*_lldA_*- and P*_lldP_-mScarlet* reporter strains in the macrocolony assay on a defined medium containing L-lactate with different amounts of added ferrous iron sulfate. We also included a strain containing the P*_A1/04/03_-mScarlet* construct; this strain constitutively synthesizes fluorescent protein and can therefore serve as a control for the effects of iron availability on overall biomass production. Additionally, to test for effects of iron availability on growth, we homogenized biofilms and plated for CFUs. As expected, biofilms grown on the medium without added iron yielded a CFU count that was approximately one order of magnitude lower than that of biofilms grown on the medium with added iron concentrations of 3.5 or 10 µM, and fluorescence levels of the P*_A1/04/03_-mScarlet* biofilms (imaged top-down on a fluorescence microscope) correlated with these relative CFU counts (Fig. 4D). We observed similar changes in fluorescence for P*_lldP_-mScarlet* biofilms. Notably, in comparison to P*_A1/04/03_-mScarlet* and P*_lldP_-mScarlet* biofilms, P*_lldA_-mScarlet* biofilms showed an inverse trend. In spite of the decreased biofilm growth yield observed on the medium without added iron, the P*_lldA_-mScarlet* reporter biofilm showed a significant increase in fluorescence in this medium when compared to the 3.5-µM-added-iron condition. Additionally, although growth yields were similar with 3.5 µM- and 10 µM-added-iron, *lldA* expression was significantly lower in the high-iron condition. In summary, our experiments examining *lld* gene expression in liquid cultures and biofilms indicate that iron limitation enhances the expression of *lldA*, but not *lldPDE*, consistent with the *lldA*’s chromosomal location (near iron-related genes). In biofilm experiments specifically, we also observed that the addition of excess iron was inhibitory to *lldA* expression.

### *lldPDE* and *lldA* are differentially expressed across biofilm depth

**Fig 5:**
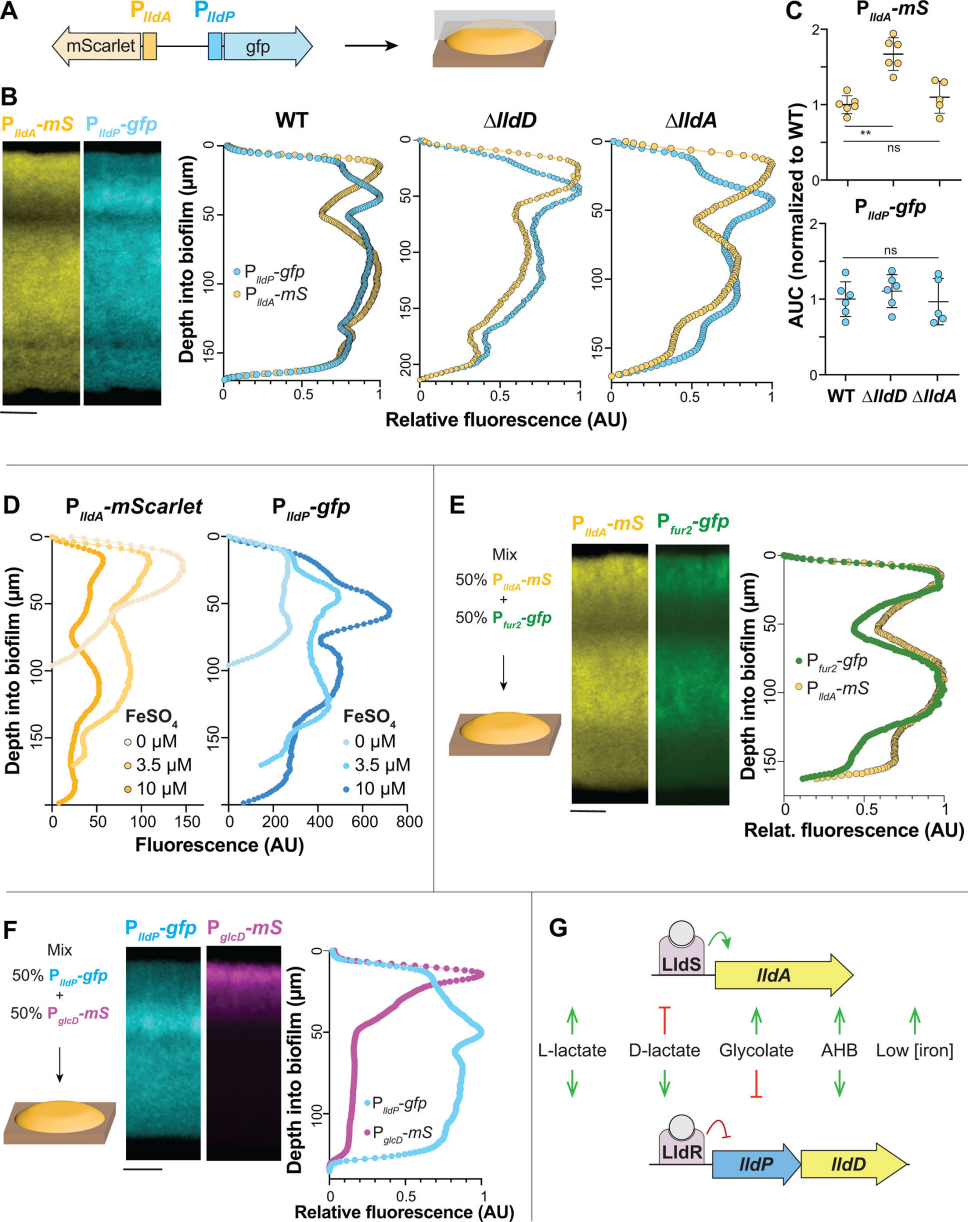
Biofilms display patterns of *lldA* and *lldPDE* expression across depth, which might arise from local differences in iron availability and glycolate production. (**A**) Schematic showing the genomic arrangement of the dual reporter construct (P*_lldA_-mScarle*t, P*_lldP_-gfp*) used in these experiments and the orientation of thin-sectioning for a macrocolony biofilm. (**B**) Left: fluorescence images of a thin-section from a biofilm formed by the dual P*_lldA_-mScarlet* (“*mS*”), P*_lldP_-gfp* reporter strain. *mScarlet* fluorescence is shown in yellow, and *gfp* fluorescence is shown in cyan. Right: fluorescence across biofilm depth for the P*_lldA_* and P*_lldP_* reporters in the indicated strain backgrounds. Images and quantification are representative of at least three biological replicates. (**C**) Quantification of total dual-reporter thin-section fluorescence expressed as the area under the curve (AUC) for *lldA* (top) and *lldPDE* (bottom) reporters in WT, ∆*lldD*, and ∆*lldA*, normalized to average wild-type fluorescence. Each dot is representative of a single biological replicate, and error bars represent standard deviation. **P < 0.01, ns = not significant. (**D**) Fluorescence across biofilm depth for each reporter in biofilms of the dual-reporter strain grown on medium amended with ferrous sulfate as indicated. Profiles are representative of three biological replicates for each iron availability condition. (**E**) Left: schematic of the experimental design for growth of biofilms containing two reporter strains: P*_lldA_-mScarlet* and P*_fur2_-gfp*. Center: fluorescence images of thin-section from a mixed biofilm. *mScarlet* fluorescence is shown in yellow, and *gfp* fluorescence is shown in green. Right: fluorescence across biofilm depth for the P*_lldA_-mScarlet* and P*_fur2_-gfp* reporters. Images and quantification are representative of four biological replicates. (**F**) Left: schematic of the experimental design for growth of biofilms containing two reporter strains: P*_glcD_-mScarlet* and P*_lldP_-gfp*. Center: fluorescence images of a thin-section from a mixed biofilm. *mScarlet* fluorescence is shown in magenta, and *gfp* fluorescence is shown in cyan. Right: fluorescence across biofilm depth for the P*_glcD_-mScarlet* and P*_lldP_-gfp* reporters. Images and quantification are representative of four biological replicates. (**G**) Visual summary of the cues that activate or inhibit the expression of *P. aeruginosa lldA* and *lldPDE*. Biofilms in panels B, D, E, and F were grown on MOPS medium containing 20 mM succinate and 10 mM L-lactate. Scale bars = 25 µm.

We predicted that the opposing gradients of oxygen (from the biofilm–air interface) and other resources (from the biofilm–medium interface) in macrocolony biofilms would differentially affect the expression of *lldPDE* and *lldA*. To examine this, we grew biofilms of the dual transcriptional reporter strain (P*_lldP_*-*gfp* P*_lldA_*-*mScarlet*) for 3 days before preparing thin sections (Fig. 5A) (42). For this experiment, the medium contained 20 mM succinate as the primary carbon source so that growth would not depend solely on LldD/A activity. Imaging via fluorescence microscopy revealed intriguing and robust patterns of *lldA* and *lldPDE* expression (Fig. 5B). Most notably, the activities of P*_lldA_* and P*_lldP_* showed an inverse relationship in the top ~60 µm of the biofilm. Specifically, we detected maximal expression of *lldA* and relatively low expression of *lldPDE* at the biofilm–air interface and maximal expression of *lldPDE* in a region where *lldA* expression shows a pronounced decline (30–40 µm from the biofilm–air interface). We observed a similar pattern for biofilms inoculated with an equal mixture of individual P*_lldP_*-*gfp* and P*_lldA_*-*mScarlet* or of individual P*_lldP_*-*mScarlet* and P*_lldA_*-*gfp* reporter strains (Fig. S6). The exclusionary patterning led us to hypothesize that LldD might negatively affect the expression of *lldA* and/or vice versa. To test this, we created mutants lacking the L-lactate dehydrogenase genes (∆*lldA* and ∆*lldD*) in the background of the dual (P*_lldP_*-*gfp* P*_lldA_-mScarlet*) transcriptional reporter strain and examined promoter activity in biofilm thin sections. We found that although overall *lldA* expression was enhanced in the ∆*lldD* mutant (Fig. 5C), the characteristic expression patterns of *lldPDE* and *lldA* were unaffected by the respective gene deletions (Fig. 5B), indicating that this tight regulatory patterning is determined by other factors.

The fact that resource gradients form within biofilms raised the question of whether iron limitation contributes to induction of *lldA* at the biofilm–air interface. We, therefore, sought to test whether iron availability influences the spatial patterning of *lldA* and *lldPDE* expression across biofilm depth. We grew the dual transcriptional reporter strain for 3 days on agar containing succinate and L-lactate and various concentrations of added iron sulfate and prepared biofilm thin sections. While we found, once again, that the general patterning of *lldA* and *lldPDE* expression was retained, we also noted that *lldA* expression increased with decreasing iron availability, specifically in the region close to the air interface. Further, overall levels of expression varied. Iron availability affected the expression levels of both *lldA* and *lldPDE*, but in opposite ways (Fig. 5D). With increasing concentrations of added iron (0 µM, 3.5 µM, and 10 µM), *lldA* expression levels decreased, while those for *lldPDE* increased (the latter may be due to a general increase in biomass, as indicated in Fig. 4D). To further investigate iron availability across biofilm depth, we created a strain that reports the expression of *fur2* (“P*_fur2_-gfp*”), which is induced by low iron conditions (36). We then grew biofilms, starting from an equal mixture of P*_fur2_-gfp* and P*_lldA_-mScarlet*, on medium containing succinate and L-lactate and found that *lldA* and *fur2* expression were aligned across biofilm depth (Fig. 5E). Together, these results suggest that iron availability is a significant parameter determining the pattern of *lldA* expression in biofilms.

While iron availability could explain some of the *lldA* expression features in biofilms, it is not responsible for the distinct decrease in *lldPDE* expression close to the air interface (Fig. 5D). We also excluded that the high *lldA* expression in this region affects *lldPDE* expression (Fig. 5B), indicating that its spatial patterning is influenced by other cues. Since we had found in our compound screen that glycolate inhibits *lldPDE* expression (Fig. 3A and C), we followed up on this lead. *P. aeruginosa* contains the *glcC* gene and the adjacent *glcDEFG* operon, which are homologous to *E. coli* genes for (i) a glycolate-sensing transcription factor that induces *glcDEFG* expression and (ii) a glycolate oxidase complex, respectively (43). We constructed a P*_glcD_-mScarlet* reporter strain and confirmed using liquid-culture experiments that its activity is induced by glycolate (Fig. S7). To test the hypothesis that glycolate contributes to the pattern of *lldPDE* expression in biofilms, we used equal mixtures of P*_glcD_-mScarlet* and P*_lldP_-gfp* to inoculate agar plates containing succinate and L-lactate (Fig. 5F). We found that *glcD* expression was highly induced specifically at the biofilm–air interface. This finding suggests a build-up of glycolate at the top of the biofilm, which could explain the decreased *lldPDE* expression in this zone.

Having shown that both iron availability and exposure to specific α-hydroxycarboxylates influence the differential regulation of *lldD* and *lldA* (Fig. 5G), we next tested the contributions of LldD and LldA during macrophage infection.

### *lldD* and *lldA* contribute to persistence of *P. aeruginosa* in macrophages

**Fig 6:**
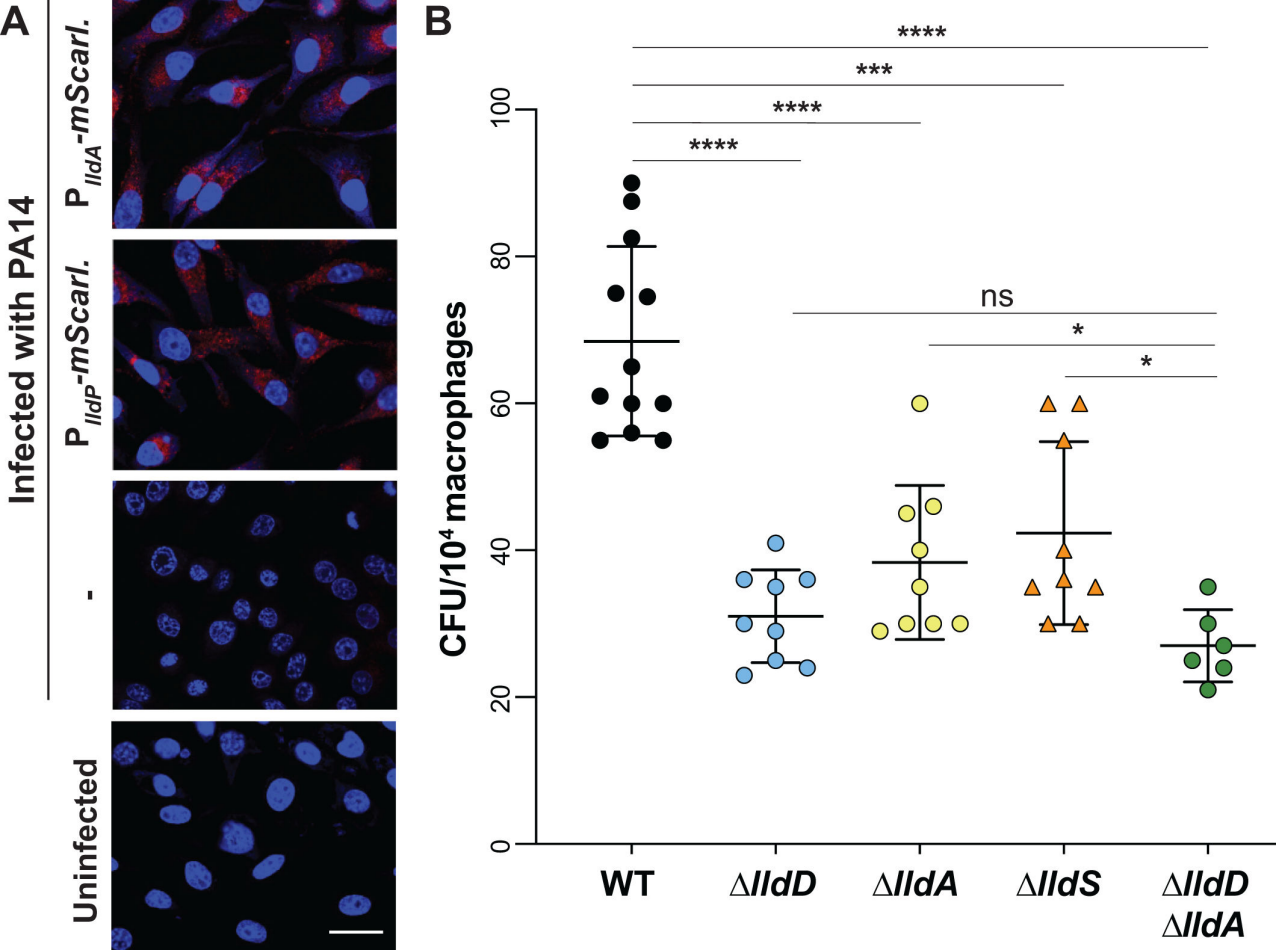
Expression of *lldA* and contributions of *lld* genes during macrophage infection. (**A**) Fluorescence images of RAW264.7 macrophages infected with the P*_lldA_-mScarlet* strain (top), the PlldP-mScarlet strain, or WT PA14, and of an uninfected control (bottom). 4′,6-Diamidino-2-phenylindole (DAPI) fluorescence is shown in blue, and mScarlet fluorescence is shown in red. Scale bar is 10 µm. (**B**) Intracellular burden of *P. aeruginosa* WT and indicated mutant strains in RAW264.7 macrophages 3 hours post-infection and subjected to the gentamicin protection assay. Each dot represents one replicate, and error bars represent standard deviation. *P < 0.05, ***P < 0.001, and ****P < 0.0001; ns = not significant.

Exposure of macrophages to bacteria or bacterial products has been reported to induce a “Warburg-like effect” in which macrophages exhibit increased glycolysis and decreased oxidative phosphorylation (44). Accordingly, studies have suggested that levels of lactate, a byproduct of glycolysis (45), are increased in infected macrophages and that lactate promotes growth of intracellular bacterial pathogens within these cells (46, 47). We therefore used RAW264.7 cells to test whether L-lactate dehydrogenase genes are expressed in the presence of macrophages and whether they contribute to *P. aeruginosa* success during infection. Macrophages incubated with the PA14 P*_lldA_-mScarlet* or P*_lldP_*-*mScarlet* reporter strains showed red fluorescence, which was absent in reporterless and uninfected controls, indicating that *lldA and lldD are* expressed during infection (Fig. 6A). To evaluate whether the L-lactate dehydrogenases contribute to *P. aeruginosa*’s success during macrophage infection, we used a gentamicin protection assay (48). We found that macrophages infected with deletion mutants lacking the L-lactate dehydrogenase genes *lldD* or *lldA* or the mutant lacking the regulator LldS showed a comparable decrease in bacterial burden when compared to those infected with WT *P. aeruginosa* (Fig. 6B). Complementation removed or mitigated this defect (Fig. S8). The observation that the double-mutant ∆*lldD*∆*lldA* phenotype is similar to the individual L-iLDH mutants suggests that the contributions of LldD and LldA are not additive under this condition. Nevertheless, these results indicate that both LldD and LldA contribute to *P. aeruginosa* persistence within macrophages.

## DISCUSSION

Enzymes that convert L-lactate to pyruvate allow bacteria to directly link a common carbon source to central metabolism. They contribute to growth alongside fermentative bacteria and eukaryotes and during colonization of eukaryotes. Within the human body, for example, L-lactate has been shown to accumulate to 1.5–3 mM in blood and tissue under normal physiological conditions and up to 40 mM under inflammatory or cancerous conditions (45), making it a significant carbon source for pathogens. Studies in various bacteria, including *Neisseria gonorrhoeae*, *Staphylococcus aureus*, and *Mycobacterium tuberculosis*, support the idea that L-lactate utilization contributes to success in the host and/or to virulence (46, 49–53). Coincidentally, all of these organisms have the capacity to produce multiple L-lactate dehydrogenases.

*P. aeruginosa* is a biofilm-forming, opportunistic pathogen that harbors traits similar to those of the nonpathogenic members of the *Pseudomonas* genus in addition to adaptations that allow it to cause disease in diverse hosts, including humans. These include the ability to grow at high temperatures and the production of a broad array of virulence factors (54–56). We were intrigued by *P. aeruginosa*’s possession of a redundant L-lactate dehydrogenase gene and the fact that this gene lies adjacent to the “pvd region” of the chromosome (36, 57), which contains multiple genes involved in iron acquisition and the response to iron limitation (Fig. 4C). This close proximity of the *lldS–lldA* locus and iron-related genes is unique to *P. aeruginosa* among the pseudomonad genomes surveyed by us. In our prior work, we showed that both of *P. aeruginosa*’s genes for L-iLDH enzymes*—lldD* and *lldA*—are expressed in liquid cultures in media containing L-lactate, including artificial sputum media, which contain this carbon source at millimolar concentrations. In liquid batch cultures, *lldD* is expressed before *lldA* and is sufficient to support wild-type growth dynamics (14). However, transcriptomic studies have shown that *lldA* is specifically induced in infection models and under infection conditions (20, 58, 59), which indicates an important role for LldA during association with hosts. We therefore investigated the regulation of *P. aeruginosa*’s *lldD* and *lldA* genes, the pattern of their expression in biofilms, and their contributions to host cell infection.

In this study, we confirmed that *lldD* expression is induced by both isomers of lactate (Fig. 3B), which alleviate repression by LldR (23). We discovered that *lldA* induction is controlled by the activator LldS (Fig. 2), which is encoded by an adjacent gene and responds specifically to the L- isomer. The chromosomal location of *lldA* led us to hypothesize that its expression could be affected by iron availability, and we indeed observed an inverse relationship between gene expression and iron availability that was unique to this L-lactate dehydrogenase gene (Fig. 4C and D). We also found that the expression of *lldA* correlates with the expression of *fur2* (Fig. 5E), a putative regulatory gene located next to *lldS* that has previously been shown to respond to iron limitation (36, 37). Moreover, we observed reduced *lldA* expression for mutants lacking *fur2* and *pvdS* (Fig. 4B), which codes for a sigma factor that controls a large regulon of genes induced by iron limitation, including *fur2* (36). We hypothesize that in response to low iron availability, PvdS increases *fur2* transcription, and Fur2 acts to increase *lldA* transcription in the presence of L-lactate, either by directly binding the *lldA* promoter or through increasing the expression of *lldS*. Iron limitation may act as a proxy signal for conditions in infection sites, where host metabolites act to sequester iron and decrease its availability for pathogens (60, 61), and where L-lactate may be available as a carbon source. Accordingly, in *N. gonorrhoeae*, it has also been suggested that the induction of an L-lactate dehydrogenase gene by iron limitation is an adaptation that promotes this pathogen’s utilization of L-lactate during infection (52). Another notable aspect of the iron-responsive regulation of *lldA* is that *P. aeruginosa*’s other L-lactate dehydrogenase gene, *lldD*, is cotranscribed with *lldE*. LldE codes for a multidomain D-lactate dehydrogenase that is predicted to contain Fe–S clusters (62). By inducing *lldA*, as opposed to *lldPDE*, under low-iron conditions, *P. aeruginosa* poises itself to utilize L-lactate but avoids production of a costly, iron-containing, and potentially superfluous enzyme.

In addition to the effect of iron availability, we evaluated the effects of a suite of metabolites on P*_lldP_* and P*_lldA_* reporter strains and found that two α-hydroxycarboxylates, α-HB and glycolate, affected the expression of one or both of the *lldPDE* and *lldA* loci. α-HB induced the expression of both *lldPDE* and *lldA* but was a more potent inducer of *lldA*. The α-HB-dependent induction of *lldA* was also dependent on LldS, suggesting that this regulator may recognize α-HB. The notion that L-lactate-binding sites can also accommodate α-HB is consistent with the structural similarity of these two compounds and the fact that studies of two L-lactate dehydrogenase enzymes from *P. stutzeri* strains have been shown to oxidize α-HB (63, 64). Little is known about the abundance of α-HB, so the physiological significance of its effects on L-lactate dehydrogenase expression and its oxidation by these enzymes is unclear.

Glycolate had an inhibitory effect on gene expression, and this effect was specific to *lldPDE*. This compound, which is also structurally similar to lactate, is found in diverse environments as it is present in large quantities in aquatic settings (65, 66) and has also been detected in chronic pressure ulcer wounds (67). In a study examining the transcriptomes of dual-species biofilms, the *P. aeruginosa glc* genes as well as *lldA* were induced during coculture with *S. aureus* when compared to monoculture conditions (68), suggesting that *S. aureus* produces both glycolate and L-lactate. In addition to these exogenous sources of glycolate, metabolic pathways within *P. aeruginosa* have the potential to produce this compound. A metabolome analysis of *P. aeruginosa* PAO1 detected enhanced glycolate levels under conditions that stimulated flux through the glyoxylate shunt, indicating that glycolate may be a byproduct of this pathway (69). Another potential source of endogenous glycolate is detoxification pathways for glyoxals, which are toxic side-products of metabolic processes and are often associated with oxidative stress (70). In organisms where glyoxal detoxification has been studied, it has been found to occur via conversion of glyoxal into glycolate by the enzymes glyoxalase I and II. *P. aeruginosa* contains homologs to these enzymes and therefore has the potential to carry out this pathway (71). Our results, which indicate that glycolate production is localized to the biofilm–air interface of macrocolonies, are consistent with both potential sources of glycolate because this region is subject to carbon limitation (a condition that promotes use of the glyoxylate shunt) and oxidative stress (a condition that would be expected to correlate with glyoxal production and detoxification) (72–75).

In previous studies examining the physiological heterogeneity and architecture of *P. aeruginosa* macrocolonies, we have used stimulated Raman scattering microscopy to detect metabolic activity (76) across biofilm depth (19). Our results show that metabolic activity levels are spatially heterogeneous in biofilms. They also show that in biofilms grown on complex or defined media and with a range of carbon source concentrations, maximum metabolic activity occurs at a distance from the biofilm–air interface (19, 75, 77). This suggests that the hypoxic biofilm subzone is more conducive to metabolism than the oxic region and raises the possibility that the redundant L-iLDH enzymes are optimized to function in, and may play a role in determining, the different levels of metabolic activity occurring in biofilm subzones. Interestingly, despite the marked differences we observed in the responses of *lldD* and *lldA* to metabolic cues, we found that both loci contribute to persistence within macrophages, indicating that while multicellular growth can promote differential use of LldD and LldA, an *in vivo* condition has the potential to promote their simultaneous use. Together, our observations provide a case study of the conditional use of redundant enzymes and provide insight into the metabolic strategies employed by a devastating bacterium that forms multicellular structures and survives intracellularly during infection (78–82).

## MATERIALS AND METHODS

### Bacterial strains and culture conditions

*Pseudomonas aeruginosa* strain UCBPP-PA14 (“PA14”) was used for all experiments. Overnight cultures were grown in lysogeny broth (LB) (83) shaking at 200 rpm at 37°C for 16–18 hours.

### Construction of markerless deletions and reporter strains

To make deletion strains, approximately 1 kb of the flanking sequence from each side of the target locus was amplified using the primers listed in Table S3 and inserted into pMQ30 through gap repair cloning in *Saccharomyces cerevisiae* InvSc1. Plasmids used in this study are listed in Table S2. Each plasmid was transformed into *Escherichia coli* strain UQ950, verified by sequencing, and moved into *P. aeruginosa* PA14 using biparental conjugation via the *E. coli* donor strain BW29427. PA14 single recombinants were selected on LB agar plates containing 70 µg/mL gentamicin. Double recombinants (markerless mutants) were selected on a modified LB medium (containing 10%[wt/vol] sucrose and lacking NaCl) containing 1.5% agar, and genotypes were confirmed by PCR. Combinatorial mutants were constructed by using single mutants as hosts for biparental conjugation, as indicated in Table S1.

To construct reporter strains, promoter regions of varying lengths were amplified from the PA14 genome using primers listed in Table S3 and inserted upstream of the coding sequence of *gfp, mScarlet,* or *luxCDABE* on their respective plasmids via ligation. Plasmids were transformed into *E. coli* UQ950 cells and verified by sequencing. Verified plasmids were introduced into PA14 using biparental conjugation with *E. coli* S17-1. Single recombinants were selected on agar plates with M9 minimal medium (47.8 mM Na_2_HPO_4_7H_2_O, 2 mM KH_2_PO_4_, 8.6 mM NaCl, 18.6 mM NH_4_Cl, 1 mM MgSO_4_, 0.1 mM CaCl_2_, 20 mM sodium citrate dihydrate, 1.5% agar) containing 70 µg/mL gentamicin or 150 µg/mL tetracycline for the luciferase reporters. The plasmid backbone was resolved out of PA14 using Flp-FRT recombination using the pFLP2 plasmid (84) and selection on M9 minimal medium 1.5% agar plates containing 300 µg/mL carbenicillin. Strains were cured of the pFLP2 plasmid by streaking on LB agar plates without NaCl and with 10% (wt/vo)l sucrose. The presence of *gfp, mScarlet,* or *luxCDABE* in final clones was confirmed by PCR.

### Liquid culture growth assays

Biological triplicates of overnight pre-cultures were diluted 1:100 in 200 µL MOPS medium (50 mM MOPS, 43 mM sodium chloride, 93 mM ammonium chloride, 2.2 mM monobasic potassium phosphate, 1 mM magnesium sulfate) containing carbon sources as indicated, in a flat-bottomed, black polystyrene 96-well plate (Greiner Bio-One 655001). Ferrous sulfate was added, at a concentration of 1 µg/mL (3.5 µM), to the MOPS medium, unless otherwise indicated. Carbon sources (succinate, lactate, glycolate, etc.) were added as sodium salts at the concentration indicated. Plates were incubated at 37°C with continuous shaking on the medium setting in a Biotek Synergy H1 plate reader. Expression of mScarlet, GFP, or luciferase was assessed by taking fluorescence or luminescence readings every 30 minutes for up to 24 hours. mScarlet was detected at excitation and emission wavelengths of 569 nm and 599 nm, respectively. GFP was detected at excitation and emission wavelengths of 480 nm and 510 nm, respectively. Growth was assessed by taking OD readings at 500 nm simultaneously with the fluorescence/luminescence readings. Unless otherwise stated, the reported values were obtained by first subtracting the fluorescence values of “no-reporter” strains and then normalizing to growth (OD at 500 nm).

### Biolog metabolite screens

Phenotypic microarray plate PM1 (Biolog, Cat. No. 12111) contains a unique carbon source in each well of a 96-well plate (File S2). For activator screens, 100 µL of MOPS medium containing 20 mM sodium succinate was added to each well of this phenotypic microarray plate, and for inhibitor screens, 100 µL of MOPS medium containing 20 mM sodium succinate and 5 mM sodium L-lactate was added to each well. Plates were incubated with shaking at 37°C for approximately 1 hour to fully dissolve each compound. The compound solutions were then transferred to a black 96-well plate pre-filled with 100 µL of the base medium per well so that the total volume per well was 200 µL. Overnight pre-cultures were diluted 1:100 into each well. Plates were placed in a Biotek Synergy H1 plate reader, and experimental parameters were as described above. A compound was considered a “hit” if the raw fluorescence value in its well (at 7.5 hours of incubation; representing the early stationary phase) was two standard deviations above (for activators) or below (for inhibitors) the median raw fluorescence value for all wells. Each screen was performed in two independent experiments, and only metabolites that were identified as hits in both experiments are reported.

### Titration experiments with luciferase reporters

The same protocol was used as for liquid culture growth assays, but with different concentrations of L-lactate, α-HB, or ferrous sulfate used to supplement the MOPS base medium. The L-lactate and α-HB titrations were performed in MOPS medium containing 20 mM succinate with L-lactate concentrations ranging from 0.1 µM to 30 mM and α-HB concentrations from 10 µM to 30 mM. The ferrous sulfate titration was done in MOPS containing 40 mM L-lactate, with the concentration of added ferrous sulfate ranging from 5 nM to 1 µM. Plates were placed in a Biotek Synergy H1 plate reader, and experimental parameters were the same as described above. Cultures were grown for 15–24 hours, and the maximum luminescence value was identified for each concentration, then normalized to growth, and to the luminescence values recorded in a promoterless *lux* strain. For lactate and α-HB titrations, this value was also normalized by subtracting the maximum fluorescence value recorded during the full incubation period in the base medium (i.e., without added L-lactate/α-HB). Where indicated, the maximum luminescence value for each L-lactate/α-HB concentration is expressed as its proportion of the maximum value exhibited for that strain at any concentration. For P*_lldP_-lux*, the maximum luminescence value was detected during growth in 500 µM L-lactate, while for P*_lldA_-lux*, the maximum luminescence value was detected during growth in 30 mM L-lactate or 30 mM α-HB (the highest concentrations tested).

### AlphaFold-based modeling

AlphaFold-Multimer (85) was used to predict models of an LldS dimer, which were manually examined with PyMOL and Coot (86). For the model of the L-lactate complex, a search for structural homologs of LldS in the Protein Data Bank (PDB) was carried out with the program Dali (87). The structure of the AmpR effector binding domain in complex with a peptide ligand (PDB entry 4WKM) (29) was found to be a close homolog, and the C-terminal Ala residue of the peptide ligand was used as the starting point for modeling L-lactate. The hydrogen-bonding interactions between the carboxylate group of Ala and the protein was maintained for L-lactate, while the position of the rest of the compound was adjusted manually. The Thr and Tyr residues in AmpR that are hydrogen-bonded to the C-terminal carboxylate of the peptide are conserved in LldS, and these residues have the same conformation in the two structures.

### Protein expression and purification

PA14 *lldS* was cloned into the pET28a vector with an N-terminal 6×His-tag. The proteins was overexpressed in *E. coli* BL21 (DE3) cells at 16°C overnight. For purification, the cell pellet was resuspended and lysed by sonication in a buffer containing 20 mM Tris (pH 8.0), 500 mM NaCl, 2 mM bME, 5% (vol/vol) glycerol, and one tablet of the protease inhibitor mixture (Sigma). The cell lysate was then centrifuged at 13,000 rpm for 30 minutes at 4°C. The protein was purified from the supernatant via nickel affinity chromatography (Qiagen). The protein was further purified by a Hiload 16/60 Superdex 200 column (Cytiva) equilibrated with a buffer containing 20 mM Tris (pH 8.0), 500 mM NaCl, and 2 mM DTT. The purified protein was concentrated to 20 mg/mL, supplemented with 5% (vol/vol) glycerol, and stored at −80°C.

### Fluorescence anisotropy

Fluorophore-labeled DNA was generated using PCR amplification with a 5′FAM-labeled reverse primer (IDT) and an unlabeled forward primer and purified using the E.Z.N.A Gel Extraction Kit (Omega Bio-tek). The amplified region contained the 256-bp region directly upstream of the *lldA* start codon. Purified LldS protein and 5 nM of FAM-labeled probe were added to the reaction buffer (50 mM HEPES [pH 7.5], 30 mM KCl, 3 mM magnesium acetate, 5% glycerol) with the protein concentration ranging from 9.37 nM to 5 µM. Parallel and perpendicular polarization values were measured in a black 384-well plate using a Biotek Synergy Neo2 plate reader with excitation and emission wavelengths of 485 nm and 528 nm, respectively, and fluorescence anisotropy was calculated using the formula (parallel − perpendicular)/parallel + (2 × perpendicular) (88). Before plotting, the minimum anisotropy value was subtracted from all data points. *K*_*d*_ was calculated in GraphPad Prism by curve-fitting using a nonlinear, least squares regression model and assuming one-site, specific binding.

### Gene arrangement and association analysis

Genomic sequences (i.e., GenBank files) of 1,323 strains were downloaded from the Pseudomonas DB (https://pseudomonas.com/) (35). For *P. aeruginosa*, only one strain, PA14, was chosen, while strains from all other species were kept for analysis. This resulted in 689 strains for the search of lactate dehydrogenase-related genes. The gene arrangement search was implemented in a custom-built tool named “Locus Hunter” (https://github.com/linyc74/locus_hunter). For each gene arrangement, blastp was used to search for homologous genes (e-value = 10^−60^) (89). The interval of each homologous gene was extended by a flanking length of 5,000 bp, resulting in intervals that overlap with each other. Intervals that overlap with each other were then fused to form a gene arrangement containing genes of interest. For each species, only one representative strain was chosen; however, if two or more strains showed different gene arrangements, both were represented. These considerations reduced the number of strains to 213. For the association analysis, two gene arrangements were defined to be associated when they coexist in a given strain. The number of associations between a pair of gene arrangements is the number of strains harboring both arrangements.

### Phylogenetic analysis

Out of the 213 strains identifed in the gene arrangement analysis (described above), 179 strains contained *lldD* and *lldA* homologs. In Geneious Prime (Version 2024.0.3), the corresponding LldD and LldA protein sequences were aligned using Clustal Omega, and a tree was constructed using Geneious Tree Builder (Jukes-Cantor; neighbor-joining; no outgroup).

### Preparation of *P. aeruginosa* macrocolony biofilms

Overnight *P. aeruginosa* cultures were subcultured at a ratio of 1:100 in 3 mL LB and were grown at 37°C, 200 rpm to an OD_500_ of ~0.5–0.6. Then, 5 µL of liquid subcultures was spotted onto 1% agar plates (100 mm x 15 mm, Simport Scientific D210-16) containing 60 mL of MOPS medium with 20 mM sodium succinate and 10 mM sodium L-lactate. For biofilms used for thin-sectioning experiments, agar plates were prepared in two layers: a 45-mL base layer and a 15-mL top layer. The base MOPS medium was prepared the same in every case, except in experiments testing the effect of iron availability, in which ferrous sulfate was omitted for the “0 µM” condition and added in excess for the “10 µM” condition. For mixed biofilms containing two reporter strains, the OD_500_ of each subculture was corrected to exactly 0.5, and subcultures were mixed in a 1:1 ratio before being spotted. Macrocolony biofilms were grown in the dark at 25°C. Macrocolony biofilm experiments were conducted with at least three biological replicates of each strain and condition.

### Top-down fluorescence imaging of *P. aeruginosa* macrocolony biofilms

After 3 days of growth as described above, bright-field and fluorescence images were obtained using a Zeiss Axio Zoom V16 fluorescence stereo zoom microscope (excitation, 545 nm; emission, 605 nm for imaging of mScarlet). Images were processed using the Zeiss Zen software and analyzed using Fiji/ImageJ.

### Quantification of colony-forming units (CFUs) from macrocolony biofilms

After 3 days of growth as described above, biofilms were homogenized in 1 mL PBS using a bead mill homogenizer (Omni [Kennesaw, GA] Bead Ruptor 12; at high setting for 90  s) and 0.5 g ceramic beads (Thermo Fisher 15 340 159, diameter of 1.4  mm). The cell suspension was serially diluted in PBS, plated on 1% tryptone, 1.5% agar plates and incubated at 37°C for 16 hours before CFU counting.

### Thin sectioning of *P. aeruginosa* macrocolony biofilms

After 3 days of growth on bilayer agar plates as described above, biofilms were overlaid with 15 mL 1% agar and sandwiched biofilms were lifted from the bottom agar layer and fixed in 4% paraformaldehyde in PBS at room temperature for 24 hours. Fixed biofilms were processed, infiltrated with wax, sectioned in 10-μm-thick sections, and collected onto slides as described in reference 77. Slides were air-dried overnight, heat-fixed on a hotplate for 30 minutes at 45°C, and rehydrated in the reverse order of processing. Rehydrated colonies were immediately mounted in Tris-buffered DAPI:fluorogel (Electron Microscopy Sciences) and overlaid with a coverslip. Thin sections were imaged using a Zeiss Axio Zoom V16 fluorescence stereo zoom microscope, at the same settings as described above. Images were processed using the Zeiss Zen software and analyzed using Fiji/ImageJ.

### Assessment of intracellular bacterial load in macrophages

RAW264.7 macrophage cells were plated overnight on six-well plates. Bacterial strains (WT, ∆*lldD*, ∆*lldA*, ∆*lldS*, and ∆*lldD*∆*lldA*) were grown in LB to an OD_600_ of 2.0 and then washed with PBS. Macrophages were washed with antibiotic-free Dulbecco’s modified Eagle medium (DMEM) and infected with bacteria at 5 MOI for 30 minutes at 37°C in 5% CO_2_. Bacteria that were unbound to macrophages were removed by one wash with cold DMEM, and to remove any excess extracellular bacteria, 100 µg/mL of gentamicin was added for 30 minutes; cells were washed with DMEM and transferred to the medium without gentamicin and incubated at 37°C in 5% CO_2_ for 3 hours. Infected RAW cells were lysed in distilled water. The lysed cells were immediately diluted in PBS and plated on LB agar plates to assess the intracellular bacterial load in macrophages. Bacterial CFUs were counted after incubating the plates overnight at 37°C.

### Fluorescence imaging of macrophages

RAW264.7 cells were seeded at 1 × 10^5^ cells per well on three-well-chambered coverglass slides the day prior to the experiment. Cells were infected with WT PA14 or PA14 bacteria carrying the P*_lldA_-mScarlet* reporter at an MOI of 5, as described in the previous section. Cells were then fixed in 4% paraformaldehyde in PBS (pH 7.4) for 15 minutes at room temperature. Slides were washed in 1× PBS (pH 7.4). Cells were stained with DAPI (1:10,000) at room temperature and washed in 1× PBS (pH 7.4) three times for 5 minutes each. The cells were examined using a confocal microscope (Nikon ECLIPSE Ti2; Nikon Instruments Inc., Tokyo) at 400× optical magnification. The assay was conducted in triplicate and repeated three times.

## SUPPLEMENTAL MATERIAL

File S1 mbio.00852-24-s0001.pdf Phylogram.

File S2 mbio.00852-24-s0002.csv List of all the metabolites present in Biolog's PM1 plate and their corresponding well number.

Supplemental figures mbio.00852-24-s0003.pdf Figures S1-S8.

Supplemental Tables mbio.00852-24-s0004.pdf Tables S1-S3. Strains, plasmids, primers.

ASM does not own the copyrights to Supplemental Material that may be linked to, or accessed through, an article. The authors have granted ASM a non-exclusive, world-wide license to publish the Supplemental Material files. Please contact the corresponding author directly for reuse.
