# Supplementary material for: The L-lactate dehydrogenases of Pseudomonas aeruginosa are conditionally regulated but both contribute to survival during macrophage infection
Source: mBio. 2024 Aug 20;15(9):e00852-24. doi: 10.1128/mbio.00852-24 (PMC11389411; doi:10.1128/mbio.00852-24)

Supplemental File 1. Pseudomonad L-iLDH profiles. Left: Phylogram representing the relationships between LldD and LldA proteins produced by each of the indicated strains. Center and right: L-iLDH profiles of each of the listed strains. Color shading corresponds to the genomic arrangements shown in the key at the top and in Figure 4A.

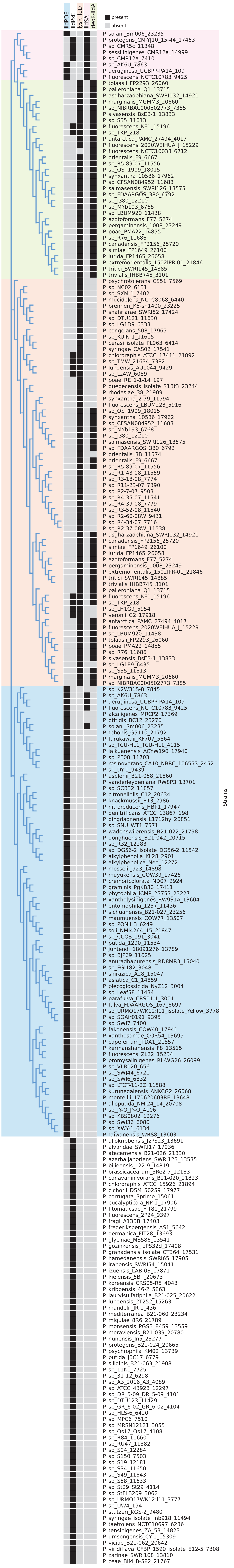

Supplement: File S1 — Phylogram. [file mbio.00852-24-s0001.pdf]
