# Supplementary material for: The L-lactate dehydrogenases of Pseudomonas aeruginosa are conditionally regulated but both contribute to survival during macrophage infection
Source: mBio. 2024 Aug 20;15(9):e00852-24. doi: 10.1128/mbio.00852-24 (PMC11389411; doi:10.1128/mbio.00852-24)
Supplement: Supplemental Tables — Tables S1-S3. Strains, plasmids, primers. [file mbio.00852-24-s0004.pdf]

Table S1. Bacterial strains used in this study.

Number	Strain	Description	Source
***Pseudomonas aeruginosa* strains**			
LD0	UCBPP-PA14 (WT)	Clinical isolate UCBPP-PA14.	(1)
LD2798	PA14 *attB*::P_{lldP}-*gfp*	PA14 containing a construct in the *attB* site that expresses *gfp* under control of the 324 bp region upstream of *lldP* (*PA14_63080*). Made using pLD2797.	(2)
LD2868	PA14 *attB*::P_{lldA}-*gfp*	PA14 containing a construct in the *attB* site that expresses *gfp* under control of the 356 bp region upstream of *lldA* (*PA14_33860*). Made using pLD2867.	(2)
LD4472	PA14 *attB*::P_{lldP}-*lux*	PA14 containing a construct in the *attB* site that expresses the *luxCDABE* operon under control of the 324 bp region upstream of *lldP* (*PA14_63080*).	This study
LD4768	PA14 *attB*::P_{lldA}-*lux*	PA14 containing a construct in the *attB* site that expresses the *luxCDABE* operon under control of the 356 bp region upstream of *lldA* (*PA14_33860*).	This study
LD4486	PA14 *attB*::MCS-*lux*	PA14 containing a construct in the *attB* site that expresses the *luxCDABE* operon with no upstream promoter. Made using pLD4440.	This study
LD4157	PA14 *attB*::P_{lldP}-*mScarlet*	PA14 containing a construct in the *attB* site that expresses *mScarlet* under control of the 324 bp region upstream of *lldP* (*PA14_63080*). Made using pLD4111.	This study
LD3760	PA14 *attB*::P_{lldA}-*mScarlet*	PA14 containing a construct in the *attB* site that expresses *mScarlet* under control of the 356 bp region upstream of *lldA* (*PA14_33860*). Made using pLD3738.	This study
LD4189	PA14 *attB*::P_{lldA}-*mScarlet,* P_{lldP}-*gfp*	PA14 containing a dual reporter construct in the *attB* site that expresses *mScarlet* under control of the 356 bp region upstream of *lldA* (*PA14_33860*) and *gfp* under control of the 324 bp region upstream of *lldP* (*PA14_63080*). Made using pLD4165.	This study
LD4187	Δ*lldD*	PA14 with *lldD* (*PA14_63090*) deleted. Made by mating pLD4132 into UCBPP-PA14.	This study
LD4052	Δ*lldA*	PA14 with *lldA* (*PA14_33860*) deleted. Made by mating pLD2758 into UCBPP-PA14.	(2)
LD3714	Δ*lldS*	PA14 with *lldS* (*PA14_33840*) deleted. Made by mating pLD3690 into UCBPP-PA14.	This study
LD4862	Δ*lldD* Δ*lldA*	PA14 with *lldD* (*PA14_63090*) and *lldA* (*PA14_33860*) deleted. Made by mating pLD2758 into LD4187.	This study
LD5130	Δ*lldD* Δ*lldS*	PA14 with *lldD* (*PA14_63090*) and *lldS* (*PA14_33840*)	This study

		deleted. Made by mating pLD4132 into LD3714.	
LD2735	Δ*lldD* Δ*lldE*	PA14 with *lldD* (*PA14_63090*) and *lldE* (*PA14_63100*) deleted. Made by mating pLD2734 into UCBPP-PA14.	(2)
LD3715	Δ*lldS attB*::P_{lldA}-*gfp*	Δ*lldS* containing a construct in the *attB* site that expresses *gfp* under control of the 356 bp region upstream of *lldA* (*PA14_33860*). Made by mating pLD3690 into LD2868.	This study
LD5116	Δ*lldS* *attB*::P_{lldA}-*mScarlet*	Δ*lldS* containing a construct in the *attB* site that expresses *mScarlet* under control of the 356 bp region upstream of *lldA* (*PA14_33860*). Made by mating pLD3690 into LD3760.	This study
LD3809	Δ*lldS*::*lldS*	Δ*lldS* (*PA14_33840*) strain with wild-type *lldS* complemented back into the site of deletion. Made by mating pLD3797 into LD3714.	This study
LD3810	Δ*lldS*::*lldS* *attB*::P_{lldA}-*gfp*	Δ*lldS* (*PA14_33840*) containing a construct in the attB site that expresses gfp under the control of the 356 bp region upstream of *lldA* with wild-type *lldS* complemented back into the site of deletion. Made by mating pLD3797 into LD3715.	This study
LD5161	Δ*lldS*::$LldS^{T107M}$	T107 of *lldS* (*PA14_33840*) mutated to methionine in the native locus. Made by mating pLD5150 into LD3714.	This study
LD5162	Δ*lldS*::$LldS^{T107M}$ *attB*::P_{lldA}-*gfp*	T107 of *lldS* (*PA14_33840*) mutated to methionine in the native locus. Containing a construct in the attB site that expresses gfp under the control of the 356 bp region upstream of *lldA*. Made by mating pLD5150 into LD3715.	This study
LD5169	Δ*lldS*::$LldS^{T107A}$	T107 of *lldS* (*PA14_33840*) mutated to alanine in the native locus. Made by mating pLD5173 into LD3714.	This study
LD5170	Δ*lldS*::$LldS^{T107A}$ *attB*::P_{lldA}-*gfp*	T107 of *lldS* (*PA14_33840*) mutated to alanine in the native locus. Containing a construct in the attB site that expresses gfp under the control of the 356 bp region upstream of *lldA*. Made by mating pLD5173 into LD3715.	This study
LD3508	Δ*lldR*	PA14 with *lldR* (*PA14_63070*) deleted. Made by mating pLD3512 into UCBPP-PA14.	This study
LD3516	Δ*lldR attB*::P_{lldP}-*gfp*	Δ*lldR* (*PA14_63070*) containing a construct in the *attB* site that expresses *gfp* under control of the 324 bp region upstream of *lldP* (*PA14_63080*). Made by mating pLD3512 into LD2798.	This study
LD3510	Δ*lldR attB*::P_{lldA}-*gfp*	Δ*lldR* (*PA14_63070*) containing a construct in the *attB* site that expresses *gfp* under control of the 356 bp region upstream of *lldA*. Made by mating pLD3512 into	This study

		LD2868.	
LD4654	Δ*fur2*	PA14 with *fur2* (*PA14_33830*) deleted. Made by mating pLD4660 into UCBPP-PA14.	This study
LD4655	Δ*fur2* *attB*::P_{lldA}-*mScarlet*	Δ*fur2* containing a construct in the *attB* site that expresses *mScarlet* under control of the 356 bp region upstream of *lldA* (*PA14_33860*). Made by mating pLD4660 into LD3760.	This study
LD4394	Δ*pvdS*	PA14 with *pvdS* (PA14_33260) deleted.	S. Häussler, Helmholtz
LD4557	Δ*pvdS* *attB*::P_{lldA}-*mScarlet*	Δ*pvdS* containing a construct in the *attB* site that expresses *mScarlet* under control of the 356 bp region upstream of *lldA* (*PA14_33860*). Made by mating pLD3738 into LD4394.	This study
LD3788	PA14 *attB*::P_{fur2}-*gfp*	PA14 containing a construct in the *attB* site that expresses *gfp* under control of the 390 bp region upstream of *fur2* (*PA14_33830*). Made using pLD3777.	This study
LD4953	PA14 *attB*::P_{glcD}-*mScarlet*	PA14 containing a construct in the *attB* site that expresses *mScarlet* under control of the 494 bp region upstream of *glcD* (*PA14_70690*). Made using pLD4757.	This study
LD4233	Δ*lldD* *attB*::P_{lldA}-*mScarlet,* P_{lldP}-*gfp*	Δ*lldD* containing a dual reporter construct in the *attB* site that expresses *mScarlet* under control of the 356 bp region upstream of *lldA* (*PA14_33860*) and *gfp* under control of the 324 bp region upstream of *lldP* (*PA14_63080*). Made by mating pLD4132 into LD4189.	This study
LD4235	Δ*lldA* *attB*::P_{lldA}-*mScarlet,* P_{lldP}-*gfp*	Δ*lldA* containing a dual reporter construct in the *attB* site that expresses *mScarlet* under control of the 356 bp region upstream of *lldA* (*PA14_33860*) and *gfp* under control of the 324 bp region upstream of *lldP* (*PA14_63080*). Made by mating pLD2758 into LD4189.	This study
LD5274	PA14 *attTn7*::$P_{PA1/04/03}$-*mScarlet*	PA14 containing a construct in the attTn7 site that expresses the coding region of *mScarlet* under control of the lac-derived constitutive *PA1/04/03* promoter.	This study
LD4600	PA14 *attB*::P_{lldA}*(256)-gfp*	PA14 containing a construct in the *attB* site that expresses *gfp* under control of the 256 bp region upstream of *lldA*. Made using pLD4600.	This study
LD4980	PA14 *attB*::P_{lldA}*(221)-gfp*	PA14 containing a construct in the *attB* site that expresses *gfp* under control of the 221 bp region upstream of *lldA*. Made using pLD4966.	This study
LD4982	PA14 *attB*::P_{lldA}*(188)-gfp*	PA14 containing a construct in the *attB* site that expresses *gfp* under control of the 188 bp region upstream of *lldA*. Made using pLD4967.	This study

LD5027	PA14 *attB*::P_{lldA}*(164)-gfp*	PA14 containing a construct in the *attB* site that expresses *gfp* under control of the 164 bp region upstream of *lldA*. Made using pLD5029.	This study
LD4599	PA14 *attB*::P_{lldA}*(125)-gfp*	PA14 containing a construct in the *attB* site that expresses *gfp* under control of the 125 bp region upstream of *lldA*. Made using pLD4599.	This study
LD4598	PA14 *attB*::P_{lldA}*(105)-gfp*	PA14 containing a construct in the *attB* site that expresses *gfp* under control of the 105 bp region upstream of *lldA*. Made using pLD4598.	This study
***E. coli* strains**			
LD44	UQ950 E. coli	DH5α λ(*pir*) strain for cloning; F-Δ(*argF-lac*)169Φ80d*lacZ*58 (ΔM15) *glnV44*(AS) *rfbD1 gyrA96*(NalR) *recA1 endA1 spoT thi-1 hsdR17 deoR λpir*+ D	D. Lies, Caltech
LD661	BW29427	Donor strain for conjugation: *thrB1004 pro thi rpsL hsdS lacZ* ΔM15RP4–1360 Δ(*araBAD*)*567* Δ*dapA1341*::[*erm pir*(wt)]	W. Metcalf, University of Illinois
LD69	β2155	Helper strain. *thrB1004 pro thi strA hsdsS lac*ZΔM15 (F' *lacZ*ΔM15 lacI^q *traD36 proA$^+$ proB$^+$*) Δ*dapA*::*erm* (Erm^R) *pir*::RP4 [::kan (Km^R) from SM10]	(3)
LD2901	S17-1	Str^R , Tp^R , F-RP4-2-*Tc*::*Mu aphA*::*Tn7 recA λpir* lysogen	(4)
	BL21 (DE3)	Competent *E. coli* strain used for protein purification.	New England BioLabs
***Saccharomyces cerevisiae* strains**			
LD676	InvSc1	*MATα/MATα leu2/leu2 trp1-289/trp1-289 ura3-52/ura3-52 his3-Δ1/his3-Δ1*	Invitrogen

Table S2. Plasmids used in this study.

Plasmid name	Description	Source
pMQ30	Yeast-based allelic-exchange vector; *sacB*+, CEN/ARSH, URA3+, Gm^R.	(5)
pFLP2	Site-specific excision vector with cI857-controlled FLP recombinase. encoding sequence, *sacB*+ , Amp^R . Used to insert LD2722-based plasmids into *P. aeruginosa* strains.	(6)
pLD2722	mini-CTX derived plasmid. Gm^R , Tet^R flanked by Flp recombinase target (FRT) sites to resolve out resistance cassettes. For making GFP reporters.	(7)
pLD3208	mini-CTX derived plasmid. Gm^R , Tet^R flanked by Flp recombinase target (FRT) sites to resolve out resistance cassettes. For making mScarlet reporters.	(8)
pLD4440	pMINI-lux, a mini-CTX derivative containing promoterless *luxCDABE* operon, Tet^R	(9)
pLD3433	mini-Tn7 derived plasmid. Gm^R , Cm^R $P_{PA1/04/03}$::*mScarlet*	(10)
pLD2797	324 bp upstream of *lldP* PCR fragment in pLD2722.	(2)
pLD2867	356 bp upstream of *lldA* PCR fragment in pLD2722.	(2)
pLD4111	800 bp *mScarlet-terminator* PCR fragment ligated into pLD2797 using SacI and XhoI.	This study
pLD3738	800 bp *mScarlet-terminator* PCR fragment ligated into pLD2867 using SacI and XhoI.	This study
pLD4441	324 bp upstream of *lldP* PCR fragment ligated into pLD4440 using XhoI and EcoRI.	This study
pLD4769	356 bp upstream of *lldA* PCR fragment ligated into pLD4440 using XhoI and EcoRI.	This study
pLD4165	2943 bp PCR fragment amplified from pLD3738 containing the 356 bp *lldA* promoter and *mScarlet-terminator* sequence ligated into pLD2797 using SpeI and BamHI.	This study
pLD3777	390 bp upstream of *fur2* (*PA14_33830*) PCR fragment ligated into pLD2797 using XhoI.	This study
pLD4757	494 bp upstream of *glcD* (*PA14_70690*) PCR fragment ligated into pLD3208 using SpeI and EcoRI.	This study
pLD4600	256 bp upstream of *lldA* (*PA14_33860*) PCR fragment ligated into pLD2722 using SpeI and XhoI.	This study
pLD4966	221 bp upstream of *lldA* (*PA14_33860*) PCR fragment ligated into pLD2722 using SpeI and XhoI.	This study

pLD4967	188 bp upstream of *lldA* (*PA14_33860*) PCR fragment ligated into pLD2722 using SpeI and XhoI.	This study
pLD5029	164 bp upstream of *lldA* (*PA14_33860*) PCR fragment ligated into pLD2722 using SpeI and XhoI.	This study
pLD4599	125 bp upstream of *lldA* (*PA14_33860*) PCR fragment ligated into pLD2722 using SpeI and XhoI.	This study
pLD4598	105 bp upstream of *lldA* (*PA14_33860*) PCR fragment ligated into pLD2722 using SpeI and XhoI.	This study
pLD2758	Δ*lldA* (*PA14_33860*) flanking fragments introduced into pMQ30 by gap repair cloning in yeast strain InvSc1.	(2)
pLD2734	Δ*lldDE* (*PA14_63090-63100*) flanking fragments introduced into pMQ30 by gap repair cloning in yeast strain InvSc1.	(2)
pLD4132	Δ*lldD* (*PA14_63090*) flanking fragments introduced into pMQ30 by gap repair cloning in yeast strain InvSc1.	This study
pLD3690	Δ*lldS* (*PA14_33840*) flanking fragments introduced into pMQ30 by gap repair cloning in yeast strain InvSc1.	This study
pLD4660	Δ*fur2* (*PA14_33830*) flanking fragments introduced into pMQ30 by gap repair cloning in yeast strain InvSc1.	This study
pLD3512	Δ*lldR* (*PA14_63070*) flanking fragments introduced into pMQ30 by gap repair cloning in yeast strain InvSc1.	This study
pLD3797	The CDS of *lldS* with ~1 kb flanks on either side, introduced into pMQ30 by gap repair cloning in yeast strain InvSc1.	This study
pLD5150	The CDS of *lldS* with ~1 kb flanks on either side with T107 changed to an methionine, introduced into pMQ30 by gap repair cloning in yeast strain InvSc-1.	This study
pLD5173	The CDS of *lldS* with ~1 kb flanks on either side with T107 changed to an alanine, introduced into pMQ30 by gap repair cloning in yeast strain InvSc-1.	This study
pET28a	Expression plasmid used for protein purification. Contains a T7 promoter, 6x-His tag. Kan^R	Addgene
pET28a-lldS	Expression plasmid containing a T7 promoter in front of the *lldS* sequence. Contains an N-termin 6x-His tag. Kan^R	This study

Table S3. Primers used in this study.

Primer Number	Sequence
Primers for plasmids pLD4111, pLD3738 (used to make P_{lldP}-*mScarlet,* P_{lldA}-*mScarlet*)	
2609	acgtacgt ctcgag tctagatttaaga aggaga tatacat ATGAGTAAAGGAGAAGC
2635	actgactg gagctc ATAAAACGAAAGGCCCAGTCTTTCG
Primers for plasmid pLD4441 (used to make P_{lldP}-*lux*)	
3895	tatctcgaggctCGACACCCTTACCCGAAGTT
3896	tatgaattcgttGGGTTGGCTCCCTAATTGTTG
Primers for plasmid pLD4769 (used to make P_{lldA}-*lux*)	
4121	tatctcgaggctTGCTCGATTTGGGCATGAC
4122	tatgaattcgttGCAGTCCACTCCTTCGGG
Primers for plasmid pLD4165 (used to make dual reporter P_{lldP}-*gfp*, P_{lldA}-*mScarlet*)	
3781	catagactagtctatgcgcggtcgatgcgatgtcgatagcTGCTCGATTTGGGCATGACC
3783	catatagggatccatcgtccgacgAAGATCCCCTGATTCCCTTTGT
Primers for plasmid pLD3777 (used to make P_{fur2}-*gfp*)	
3269	ccccgggctgcaggaattccGGCACCAGCTACATCCAAC
3270	ttgtaccgggcccaagcttcCGTGACGCTCCTTTCGTG
Primers for plasmid pLD4757 (used to make P_{glcD}-*mScarlet*)	
4449	acgtacgtacactagtTCGAGCAGGTCGTAGAGGGT
4450	acgtacgtacgaattcGGCGGCTGTCCTTGTTGTG
Primers for plasmid pLD4132 (Δ*lldD*)	
3710	aggcaaattctgttttatcagaccgcttctgcgttctgatAAACCTTTCAAGGCCCTGTT
3711	ttcgatcagttcgagcaggttCAGGGTGTACTCGGCGTA
3712	tacgccgagtacaccctgAACCTGCTCGAACTGATCGAA
3713	ggaattgtgagcggataacaatttcacacaggaaacagctATCAGGTGGGTGAGGATGTC
Primers for plasmid pLD3690 (Δ*lldS*)	
2986	aggcaaattctgttttatcagaccgcttctgcgttctgatCAGGAACGCATCGCGATTCC

3198	tcatgcccaaatcgagcagGCTGCCGGTGATCGACAGG
3199	cctgtcgatcaccggcagcCTGCTCGATTTGGGCATGA
2989	ggaattgtgagcggataacaatttcacacaggaaacagctCAGCGGCGCTTGATCCATT
Primers for plasmid pLD3797 (*lldS* complementation)	
2986	aggcaaattctgttttatcagaccgcttctgcgttctgatCAGGAACGCATCGCGATTCC
2989	ggaattgtgagcggataacaatttcacacaggaaacagctCAGCGGCGCTTGATCCATT
Primers for plasmid pLD5150 (LldS^{T107M} point mutation)	
4569	ccaggcaaattctgttttatcagaccgcttctgcgttctgatCGCGGGTTGCTGGCTT
4570	gcggaatcaggatctgccgggcgAACATCGGCGGCGTGC
4571	cctgcgcacgccgccgatgttCGCCCGGCAGATCCTGAT
4572	aattgtgagcggataacaatttcacacaggaaacagctCCGTGGCGATGTCCTCCA
Primers for plasmid pLD5173 (LldS^{T107A} point mutation)	
4569	ccaggcaaattctgttttatcagaccgcttctgcgttctgatCGCGGGTTGCTGGCTT
4566	cgcggaatcaggatctgccgggcgAAGGCCGGCGGCGTG
4567	cctgcgcacgccgccggccttCGCCCGGCAGATCCTGAT
4568	aattgtgagcggataacaatttcacacaggaaacagctGGCCATCTCCTGGCCGAG
Primers for plasmid pLD3512 (Δ*lldR*)	
2877	caggcaaattctgttttatcagaccgcttctgcgttctgatATGGCGCCGATCTTGAAGG
2878	ggatgtgctggttggacaccCCTCCAGTTGCGCAACGATG
2879	catcgttgcgcaactggaggGGTGTCCAACCAGCACATCC
2880	ggaattgtgagcggataacaatttcacacaggaaacagctCAGGAACTCCGGATAGCGCT
Primers for plasmid pLD4660 (Δ*fur2*)	
4109	ggcaaattctgttttatcagaccgcttctgcgttctgatAAGTCCCTTGCCCGCG
4110	ctgctcgttggccgcgaGGCCGGCCTCCTTCAA
4111	ttgaaggaggccggcctCGCGGCCAACGAGCAG
4112	aattgtgagcggataacaatttcacacaggaaacagctTGGCGGCGGCTTCCAG
Primers for pLD4600 (P_{lldA}*(256)-gfp*)	
4117	acgtacctcgagGCAGTCCACTCCTTCGGG

4120	acgtacactagtGGTGGTGCTGTTCCCGC
Primers for pLD4966 (P_{lldA}*(221)-gfp*)	
4117	acgtacctcgagGCAGTCCACTCCTTCGGG
4486	acgtacactagtACCATTACCACCTGCGCA
Primers for pLD4967 (P_{lldA}*(188)-gfp*)	
4117	acgtacctcgagGCAGTCCACTCCTTCGGG
4487	acgtacactagtCGGCCAGCCAGCGTTT
Primers for pLD5029 (P_{lldA}*(164)-gfp*)	
4117	acgtacctcgagGCAGTCCACTCCTTCGGG
4488	acgtacactagtGGCTGGCGGCGATCT
Primers for pLD4599 (P_{lldA}*(125)-gfp*)	
4117	acgtacctcgagGCAGTCCACTCCTTCGGG
4118	acgtacactagtGGGAATTCCTTCCGGCGG
Primers for pLD4598 (P_{lldA}*(105)-gfp*)	
4117	acgtacctcgagGCAGTCCACTCCTTCGGG
4119	acgtacactagtGACGACTGAAACGGCGGA
Primers for pET28a-lldS	
LldS-F	aaaacatatgctcaatcccgacgatttc
LldS-R	aaaagtcgactcagtcgtccgccgcc

Supplemental References

1. Rahme LG, Stevens EJ, Wolfort SF, Shao J, Tompkins RG, Ausubel FM. 1995. Common virulence factors for bacterial pathogenicity in plants and animals. Science 268:1899–1902.

2. Lin Y-C, Cornell WC, Jo J, Price-Whelan A, Dietrich LEP. 2018. The Pseudomonas aeruginosa Complement of Lactate Dehydrogenases Enables Use of d- and l-Lactate and Metabolic Cross-Feeding. MBio 9.

3. Dehio C, Meyer M. 1997. Maintenance of broad-host-range incompatibility group P and group Q plasmids and transposition of Tn5 in Bartonella henselae following conjugal plasmid transfer from Escherichia coli. J Bacteriol 179:538–540.

4. Simon R, Priefer U, Pühler A. 1983. A Broad Host Range Mobilization System for In Vivo Genetic Engineering: Transposon Mutagenesis in Gram Negative Bacteria. Biotechnology 1:784–791.

5. Shanks RMQ, Caiazza NC, Hinsa SM, Toutain CM, O'Toole GA. 2006. Saccharomyces cerevisiae-based molecular tool kit for manipulation of genes from gram-negative bacteria. Appl Environ Microbiol 72:5027–5036.

6. Hoang TT, Karkhoff-Schweizer RR, Kutchma AJ, Schweizer HP. 1998. A broad-host-range Flp-FRT recombination system for site-specific excision of chromosomally-located DNA sequences: application for isolation of unmarked Pseudomonas aeruginosa mutants. Gene 212:77–86.

7. Jo J, Cortez KL, Cornell WC, Price-Whelan A, Dietrich LEP. 2017. An orphan cbb3-type cytochrome oxidase subunit supports Pseudomonas aeruginosa biofilm growth and virulence. Elife 6:e30205.

8. Okegbe C, Fields BL, Cole SJ, Beierschmitt C, Morgan CJ, Price-Whelan A, Stewart RC, Lee VT, Dietrich LEP. 2017. Electron-shuttling antibiotics structure bacterial communities by modulating cellular levels of c-di-GMP. Proc Natl Acad Sci U S A 114:E5236–E5245.

9. Higgins S, Heeb S, Rampioni G, Fletcher MP, Williams P, Cámara M. 2018. Differential Regulation of the Phenazine Biosynthetic Operons by Quorum Sensing in Pseudomonas aeruginosa PAO1-N. Front Cell Infect Microbiol 8:252.

10. Dayton H, Kiss J, Wei M, Chauhan S, LaMarre E, Cornell WC, Morgan CJ, Janakiraman A, Min W, Tomer R, Price-Whelan A, Nirody JA, Dietrich LEP. 2024. Cellular arrangement impacts metabolic activity and antibiotic tolerance in Pseudomonas aeruginosa biofilms. PLoS Biol 22:e3002205.
